# Protein acylation: mechanisms, biological functions and therapeutic targets

**DOI:** 10.1038/s41392-022-01245-y

**Published:** 2022-12-29

**Authors:** Shuang Shang, Jing Liu, Fang Hua

**Affiliations:** CAMS Key Laboratory of Molecular Mechanism and Target Discovery of Metabolic Disorder and Tumorigenesis, State Key Laboratory of Bioactive Substance and Function of Natural Medicines, Institute of Materia Medica, Chinese Academy of Medical Sciences & Peking Union Medical College, 100050 Beijing, P.R. China

**Keywords:** Cancer metabolism, Molecular biology

## Abstract

Metabolic reprogramming is involved in the pathogenesis of not only cancers but also neurodegenerative diseases, cardiovascular diseases, and infectious diseases. With the progress of metabonomics and proteomics, metabolites have been found to affect protein acylations through providing acyl groups or changing the activities of acyltransferases or deacylases. Reciprocally, protein acylation is involved in key cellular processes relevant to physiology and diseases, such as protein stability, protein subcellular localization, enzyme activity, transcriptional activity, protein–protein interactions and protein–DNA interactions. Herein, we summarize the functional diversity and mechanisms of eight kinds of nonhistone protein acylations in the physiological processes and progression of several diseases. We also highlight the recent progress in the development of inhibitors for acyltransferase, deacylase, and acylation reader proteins for their potential applications in drug discovery.

## Introduction

Protein post-translational modifications (PTMs) increase the functional diversity of the proteome by the covalent addition of functional groups to proteins. Several PTMs use metabolic intermediates, modifying the epigenetic landscape, the cell signaling networks and providing elegant mechanisms to precisely govern protein function. In the early 1960s, histone acetylation was first discovered to regulate gene transcription.^1,2^ Since the first nonhistone protein p53 was found to be regulated by acetylation in the 1980s, thousands of nonhistone proteins have been identified as acylation targets. In 2009, a research group at the University of Chicago developed a powerful algorithm named PTMap to identify all possible PTMs with high confidence.^3^ Since then, the group has taken the lead and reported eight novel acylation modifications on histone lysine (K) residue by using mass spectrometry and biochemistry technologies.^4^ These modifications include crotonylation,^5^ malonylation, succinylation,^6,7^ glutarylation,^8^ β-hydroxybutyrylation,^9^ dihydroxyisobutyrylation,^10^ benzoylation,^11^ and lactylation.^12^ In addition to modifications by short-chain acylation, modifications by long-chain fatty acid acylation, such as myristoylation, palmitoylation, and prenylation were also identified in nonhistone proteins in the 1980s.^13^

**Fig. 1:**
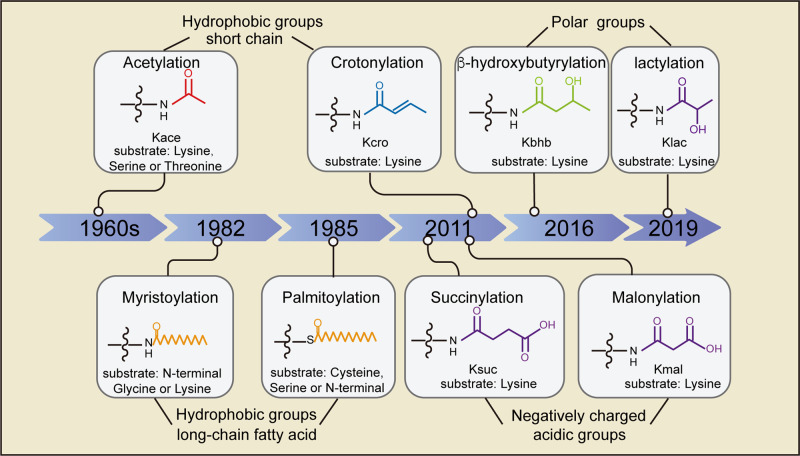
Timeline of the historical milestone for the discovery of protein acylation, and the chemical structures of acyl groups. Since acetylation was identified in 1960s, more than eight kinds of acylation modifications have been discovered, especially after 2009, because of the quick development of mass spectrometry and biochemistry technologies, as well as powerful algorithm methods. The eight kinds of protein acylations mentioned here can be divided into three groups according to their chemical structures. Acetyl- and crotonyl- are short-chain hydrophobic acyl groups. Myristoyl- and palmitoyl- are long-chain fatty acid hydrophobic acyl groups. β-hydroxybutyryl- and lactyl- belong to the polar acyl groups. Succinyl- and malonyl- belong to the negatively charged acidic acyl groups. The short-chain acylation mainly occurs at lysine residues. Whereas myristoylation often occurs at N-terminal glycine or lysine residues, and palmitoylation usually occurs at cysteine, serine or N-terminal amino acid residues

The cellular intrinsic metabolic reprogramming and the extrinsic metabolic status in the microenvironment are involved in the regulation of protein acylation. Direct participation of central metabolites in PTMs enables cells to integrate information from metabolism into complex cellular decisions that ensures proper regulation of cellular processes, such as protein stability, protein subcellular localization, enzyme activity, transcriptional activity, protein–protein interactions (PPIs) and protein–DNA interactions. In this review, we provide an overview of the expanding landscape of nonhistone protein acylation, mainly including acetylation, succinylaton, malonylation, crotonylation, β-hydroxybutyrylation, lactylation, myristoylation and palmitoylation (Fig. 1). We discuss the generation of the acyl group donor acyl-CoA, the enzymatic regulation of acylation, how the acylation marks are identified by the reader proteins, as well as the general biological function of protein acylation. Abnormal and imbalanced acylation of nonhistone protein in association with various human diseases, especially cancer will be discussed. We also provide a glimpse into the potential value of protein acylation from a therapeutic view and conclude with a discussion of key open questions and future perspectives.

## Donors, writers, erasers and readers of protein acylation

Protein acylation is regulated either in a nonenzymatic or enzymatic manner with the latter one more common. In the enzymatic dependent condition, the acyltransferase—“writer” is responsible to add acyl groups from the “donors” of acyl-CoA, including acetyl-, succinyl-, malonyl-, crotonyl-, β-hydroxybutyryl-, lactyl-, myristoyl-, and palmitoyl-CoA, to the side-chain of lysine (glycine, cysteine, serine or others) residues. The deacylase—“eraser” catalyzes the removal of acyl groups from the aforementioned amino acid residues. The acylation marks are usually read by the specific protein domains, sometimes referred to as “reader” (Table 1).

**Table 1:** Writers, readers, erasers, and donors of protein acylation

Acylation type	Writer	Reader	Eraser	Donor
Acetylation	KAT1~17: (1) p300/CBP family (p300, CBP); (2) GNAT family (GCN5, PCAF); (3) MYST family (MOZ, MOF, Sas2, Sas3, Tip60); (4) SRC family (SRC-1, 2 and 3); (5) Others (ACAT1).	BRD4, BRD3, PBRM1	HDAC family: (1) Class I (HDAC1, 2, 3 and 8); (2) Class II (IIa: HDAC4, 5, 7 and 9; IIb: HDAC6 and 10) (3) Class III (SIRT1-7); (4) Class IV (HDAC11).	Acetyl-CoA: glycolysis, lipid β-oxidation, branched amino acids, glutamine, Kbhb, acetate
Crotonylation	p300/CBP, MOF, GCN5	NA	SIRT1, SIRT2, SIRT3	Crotonyl-CoA: lysine, hydroxylysine and tryptophan, crotonate, glutaryl-CoA
Malonylation	p300/CBP, GCN5, PMAT1	NA	SIRT5	Malonyl-CoA: acetyl-CoA, malonate
Succinylation	p300/CBP, GCN5, CPT1A	NA	SIRT5, SIRT7	Succinyl-CoA: succinate, α-KG, isoleucine, methionine, and valine
β-hydroxybutyrylation	p300/CBP	NA	SIRT3, HDAC1, HDAC2	β-hydroxybutyryl-CoA: β-hydroxybutyrate
Lactylation	p300/CBP	NA	HDAC1, HDAC3	Lactyl-CoA: lactate
Myristoylation	NMT family (NMT1 and NMT2)	NA	SIRT6, IpaJ	Myristoyl-CoA: edible oil (coconut oil, butter, and palm oil)
Palmitoylation	ZDHHC family (ZDHHC1–23)	NA	APT, PPT, ABHD	Palmitoyl-CoA: edible oil (coconut oil, butter, and palm oil)

### Donors and the biological function of protein acylation

Acyl-CoA often acts as acyl donor for protein acylation, which are mainly derived from metabolites of glucose, fatty acid, and amino acid. The protein acylation level has a close relationship with cellular acyl-CoA concentration; therefore it is dynamically regulated by metabolic state such as feeding and starvation.^14^ The differences of acyl-CoA chemical structures mainly contribute to their different influences on the physiochemical properties of the substrate proteins, such as the hydrophobicity, electric charge, steric hindrance and so on. The eight kinds of protein acylation mentioned in this review can be divided into three groups according to their chemical structures: (1) hydrophobic acyl groups, including short-chain acylation of acetyl and crotonyl; long-chain lipid acylation of palmitoyl and myristoyl, which increase protein hydrophobicity and membrane binding ability. (2) Negatively charged acidic acyl groups, including malonyl and succinyl, which change the charge at the K residue from +1 to −1 without disturbing the physiological pH level.^15^ (3) Polar acyl groups, including β-hydroxybutyryl and lactyl, which contain a hydroxyl group to enable the formation of hydrogen bonds with other proteins.^4^ We will then describe the source of each kind of donor in detail.

#### Donors of protein acetylation

As the donor of protein lysine acetylation (Kace), acetyl-CoA is a central metabolite and substrate for anabolic metabolism, which has been studied thoroughly. It can be produced in the mitochondria, cytoplasm or nucleus. Mitochondrial acetyl-CoA is derived from glycolysis, lipid β-oxidation, and the catabolism of branched amino acids (i.e., valine, leucine, and isoleucine). In glycolysis, mitochondrial pyruvate is decarboxylated to form acetyl-CoA, CO_2_, and nicotinamide adenine dinucleotide hydride (NADH) by the pyruvate dehydrogenase complex (PDC).^16^ In β-oxidation, the acyl-CoA synthetase protein family catalyzes the CoA- and adenosine triphosphate (ATP)-dependent conversion of cytosolic free fatty acids into acyl-CoA.^17^ In amino acid metabolism, branched-chain amino acids are first transformed to branched-chain α-ketoacids and then catalyzed into NADH, acetyl-CoA, and other acyl-CoA thioesters.^18^ Besides the three ubiquitous metabolic circuitries mentioned above, acetyl-CoA also comes from organ-specific pathways. It could derive from β-hydroxybutyrylate in neuron or from ethanol–acetaldehyde–acetate axis in liver cells or brain cells.^19–21^ Glycolysis- or β-oxidation-derived mitochondrial acetyl-CoA represents the major source of cytosolic acetyl-CoA upon transportation. In addition, cytosolic acetyl-CoA can also be derived from glutamine reductive carboxylation, especially when glycolysis is blocked and from acetate in an ATP-dependent manner.^22,23^ The acetyl-CoA in the nucleus is freely diffused from the cytosol. Besides, two acetyl-CoA-generating enzymes, namely ATP-citrate lyase (ACLY) and acyl-CoA synthetase short-chain family member 2 (ACSS2), are localized in the nucleus and linked to cell growth and proliferation.^24,25^

#### Biological function of protein acetylation

All lysine acetyltransferases (KATs) require acetyl-CoA as donor for acetylation reactions. Reciprocally, protein lysine acetylation represents an important mechanism to regulate overall energy metabolisms through either metabolic enzymes or transcription factors. A proteomic analysis of lysine acetylation in rat islets revealed that almost all enzymes in core metabolic pathways related to insulin secretion were acetylated in response to high glucose.^26^ For example, glucose increased the acetylation of trifunctional enzyme subunit alpha (ECHA, catalyzing the second and third step of long-chain fatty acid β-oxidation) at K644 and K505. Such modifications significantly decreased fatty acid β-oxidation and enhanced the insulin secretion in islet β-cells. As one of the members of the mammalian forkhead box O (FOXO) transcription factor family, FOXO1 is highly expressed in insulin-responsive tissues, including the pancreas, liver, skeletal muscle, and adipose tissue to orchestrate energy homeostasis. FOXO1 acetylation is catalyzed by CREB binding protein (CBP) at K242, K245, and K262. The positive charge of these lysines in FOXO1 contributes to its DNA-binding activity; and acetylation at these residues reduces its binding ability to DNA sequence and attenuates its transcriptional activity.^27^ FOXO1 acetylation is regulated during the feed-fast cycles.^28^ In fasting status, hepatic ETS Proto-Oncogene 1 (ETS1) expression is suppressed through MEK-ERK pathway, allowing FOXO1 nuclear trapping and glucogenetic genes transcription. During the feeding period, elevated ETS1 cooperates with CBP to induce FOXO1 acetylation via enhancing their association. Such effect promotes FOXO1 nuclear export and suppresses hepatic gluconeogenesis. Exercise is a good way to boost metabolism. After exercise, widespread protein lysine acetylation could be observed in the skeletal muscle, which is critical for muscle contraction and structure.^29^

Protein kinases bind ATP and use it to phosphorylate other proteins. Acetylation is known to regulate kinase activity through impairing or enhancing ATP binding. Generally, there is a conserved lysine residue in the ATP-binding pocket of protein kinases, which could be acetylated to affect kinase catalytic activity. Cyclin-dependent kinase 5 (CDK5) is highly expressed in the brain and plays a role in regulating axonal and dendritic growth, neuronal migration, and synapse development. CDK5 acetylation at K33 by general control nonrepressed-protein 5 (GCN5) leads to the loss of its kinase activity via impairing the ATP binding, negatively regulating neurite outgrowth and determining neurite length.^30^ Notably, acetylation of the conserved lysine residue in ATP-binding pocket is not always impairing the ATP binding. P38 mitogen-activated protein kinase (MAPK) was reported to be reversibly acetylated by p300/CREB binding protein-associated factor (PCAF)/p300 and histone deacetylases 3 (HDAC3) at K53, a lysine site located in its ATP-binding pocket.^31^ Acetylation of K53 increases the binding affinity of p38 with ATP and enhances its kinase activity. These observations suggest that acetylation at the conserved lysine in the ATP-binding pocket could be a mechanism in controlling kinase activity. However, why lysine acetylation increases the ATP-binding activity in some kinases but decreases it in others still needs further investigation.

#### Donors of other protein acylation

The research on acyl-CoA of other short-chain acylations including lysine malonylation (Kmal), succinylation (Ksuc), crotonylation (Kcro), β-hydroxybutyrylation (Κbhb) and lactylation (Klac) or long-chain acylation including myristoylation and palmitoylation is not so thorough and enough. Until now, there are mainly three sources of acyl-CoA for these short-chain acylation: (1) short-chain fatty acid (SCFA) or carboxylic acid, including malonate, succinate, crotonate, β-hydroxybutyrate, lactate and α-ketoglutarate (α-KG). Under insufficient glucose energy supply and the fatty acid mobilization conditions, SCFA is transfered into acyl-CoA via ACSS2 to enhance lipid β-oxidation and protein acylation of malonylation, succinylation, crotonylation and β-hydroxybutyrylation. The lactyl-CoA level is upregulated in the condition of anaerobic glycolysis. Besides, succinyl-CoA is mainly derived from α-KG via the α-KG dehydrogenase complex (α-KGDHC) in the tricarboxylic acid (TCA) cycle.^32–35^ (2) Amino acids metabolism. Acyl-CoA also derived from amino acid catabolism. Isoleucine, methionine, and valine could be transformed into succinyl-CoA. Lysine, hydroxylysine, and tryptophan could be transformed into crotonyl-CoA.^36^ (3) Other kinds of acyl-CoA. Acyl-CoA for the short-chain acylation could also be produced by other acyl-CoA via carboxylases. For example, acetyl-CoA could be catalyzed by acetyl-CoA carboxylase (ACCase) into malonyl-CoA.^37^ Crotonyl-CoA could be transformed from Glutaryl-CoA by Glutaryl-CoA dehydrogenases (GDHs) through dehydrogenation and decarboxylation.^38^ In the long-chain fatty acid acylation, the myristoyl- and palmitoyl-CoA are often derived from long-chain fatty acid existed in the edible oil such as coconut oil, butter and palm oil.

#### Biological function of protein succinylation and malonylation

SIRT5 acts as desuccinylase, demalonase and deglutarylase. Sirt5^−/−^ mice were widely used to study the role of succinylation and malonylation. The three kinds of SIRT5-regulated acylations are all connected with metabolism regulation, such as fatty acid oxidation. A systematic profiling of the mammalian succinylome revealed potential impacts of lysine succinylation on enzymes involved in mitochondrial metabolism, the TCA cycle, and fatty acid metabolism.^39^ Succinate dehydrogenase (SDH) catalyzes the sixth step of TCA cycle to convert succinate into fumarate. SDH succinylation activates its enzymatic activity, suggesting a self-regulatory mechanism of succinate levels in mitochondria.^39^ Another metabolomics-assisted proteomic study identifies protein lysine succinylation predominantly accumulates in the heart when Sirt5 is deleted, suggesting succinylation in the regulation of heart metabolism and function.^40^ Here, the succinylation impairs fatty acid oxidation through downregulation of ECHA activity, resulting in lower cardiac ATP production and heart dysfunction during energy-demanding situations such as fasting and exercise. Malonyl-CoA is a tightly regulated metabolic intermediate, which is produced by acetyl-CoA carboxylase and consumed by malonyl-CoA decarboxylase (MCD), fatty acid synthase, and fatty acid elongases. Using MCD^−/−^ cells as a model, increased lysine malonylation was found to show impaired mitochondrial respiration and fatty acid oxidation.^41^ These studies indicate that succinylation or malonylation of metabolic enzymes function as a crosstalk mechanism between metabolic processes and nutrient change.

#### Biological function of protein β-hydroxybutyrylation and crotonylation

β-hydroxybutyrate (β-OHB) is the most abundant ketone body. Besides oxidation as an energy substrate, β-OHB is involved in PTMs of histone and nonhistone proteins. Starvation and ketogenic diet stimulate global protein Kbhb in the liver and kidney. Several enzymes of the methionine cycle were β-hydroxybutyrylated in the liver, suggesting that protein β-hydroxybutyrylation may play a role in methionine homeostasis under metabolic stresses, such as prolonged fasting and ketogenic diet.^42^ Different from the tissue specific distribution of β-hydroxybutyrylation, crotonylation of nonhistone protein is widely distributed in subcellular compartments and affects diversity of protein function, such as gene transcription, DNA damage response, enzymes regulation and metabolic pathways.^43^ Crotonate, mainly produced by the colon microbiota, is the SCFA precursor of crotonyl-CoA. From this aspect, crotonylation can be considered as a link between the host and gut microbiota.

#### Biological function of protein palmitoylation and myristoylation

Palmitoylation and myristoylation represent the two most common protein lipid modifications. Myristoylation is an important protein modification in the immune response catalyzed by N-myristoyltransferase (NMT). Thymus is the primary site for T-cell development and has been shown to have high NMT activities. Myristoylation is an essential lipid modification in the thymus during T-cell development.^44^ In addition, myristoylation is indispensable for the formation of immunological synapse. Myristoylation of Lck and Fyn is necessary for their localization to the immunological synapse, allowing the activation of T-cell receptor (TCR) signaling.^45^ N-myristoylation is also involved in the control of innate immunity. TRIF-related adaptor molecule (TRAM) is an adaptor molecule exclusively function in the Toll-like receptor 4 (TLR4) pathway. Myristoylation of TRAM targets it to the plasma membrane, where it is essential for the LPS-induced release of inflammatory mediators and cytokines through the TLR4 signaling.^46^ Similar to myristoylation, palmitoylation is also crucial for protein-membrane docking. For example, Src protein requires both myristoylation and palmitoylation together to form a “dual signal” motif that targets them to membranes.^47^ In fact, palmitoylation is well-known to be highly prevalent among neuronal proteins and may be relevant to the processes of learning and memory.^48,49^

### Writers of protein acylation

Writers of protein acylation mainly include KAT family, zinc finger aspartate–histidine–histidine–cysteine (DHHC)-type containing (ZDHHC) family and NMT family. KAT family was first regarded as writers of acetylation. Whereas, with the discovery of novel protein acylation types, many members of KAT family were shown to have an expanded repertoire of other short-chain acyltransferase activities. ZDHHC and NMT families are mainly responsible for palmitoylation and myristoylation.

#### Writers of protein acetylation

Approximately 17 human KATs have been identified of histone acetyltransferases (HATs), which can be divided into five families based on the degree of sequence similarity. The KATs consist of the GCN5-related N-acetyltransferases family (GNAT), which are represented by GCN5 and PCAF; the p300/CBP family, including p300 and CBP; the MYST family, which is represented by MOZ, MOF, Ybf2 (Sas3), Sas2 and TAT interacting protein 60 (Tip60), and monocytic leukemia zinc finger protein; the steroid receptor coactivator (SRC) family, which is represented by SRC-1, 2 and 3; and acetyltransferases, which can not be clearly categorized based on defining features of the first four classes, such as acetyl-CoA acetyltransferase 1 (ACAT1).

#### Writers of other short-chain protein acylation

P300/CBP is the common writer for almost all short-chain protein acylations, as it has a deep aliphatic pocket within the active site, a critical feature to bind with bulk acyl groups that is not observed in other HATs.^50^ Members of the GNAT and MYST families have more limited range of acylation activities. For example, Kcro is written by MOF and GCN5, Kmal and Ksuc are written by GCN5 besides p300.^51–55^ Except for the HATs, Kmal and Ksuc separately have their specific writers of phenolic glucoside malonyl-transferase 1 (PMAT1) and carnitine palmitoyl transferase 1A (CPT1A).^56,57^ In a study of SIRT5-regulated lysine malonylome using label free quantitative proteomics, researchers found that 56% of mitochondrial Kmal sites overlapped with previously identified Ksuc sites, and 44% of Kmal sites were distinct from both succinylation and acetylation. This is obviously distinct to the fact that succinylation sites overlap with 80% sites of other acylations. They speculated that there might be one acyltransferase utilizing both acetyl-CoA and succinyl-CoA while another acyltransferase possessing high selectivity of malonyl-CoA. It is rational to guess that many specific writers are still waiting to be found behind the “acylation code”.^58^ Notably, many acylations are regulated both in nonenzymatic and enzymatic manners such as Kace, Ksuc, and Kcro. The nonenzymatic process is mainly controlled by the concentration of acyl-CoA in mitochondria, pH, and protein parameters_._^59,60^

#### Writers of protein myristoylation and palmitoylation

In contrast to the lysine residue-mediated post-translational modifications mentioned above, protein myristoylation mainly occurs on the N-terminal glycine or lysine residues via a stable amidic linkage catalyzed by NMT1 and NMT2. Protein palmitoylation is mainly categorized into S-palmitoylation (cysteine residues, Spalm), O-palmitoylation (serine residues), and N-palmitoylation (N-terminus).^61^ Spalm is mediated by the palmitoyl-acyltransferases (PAT) of ZDHHCs, which contain 23 distinct members in mammals. Mechanistically, the cysteine residue of the DHHC domain reacts with palmitoyl-CoA, forming an acyl intermediate, and then transfers it directly to the substrate protein.

### Erasers of protein acylation

#### Erasers for protein acetylation

HDACs are enzymes that catalyze the removal of acetyl functional groups from the lysine residues of both histone and nonhistone proteins. HDACs can be divided into two categories, Zn^2+^-dependent and NAD^+^-dependent HDACs. Zn^2+^-dependent HDACs include class I (HDAC1, 2, 3, and 8), II (IIa: HDAC4, 5, 7, and 9; IIb: HDAC6 and 10) and IV (HDAC11) subgroups, while NAD^+^-dependent enzymes are class III HDACs (SIRT1-7).^62–64^

#### Erasers for other short-chain protein acylation

The short-chain protein acylation share the deacylases of HDAC I, II, and III proteins due to their similar chemical structures of amide bond. The removal of protein Kmal and Ksuc, the negatively charged acidic acyl groups, is mainly dependent on SIRT5, which is closely related to some diseases of cancer and neurodegenerative diseases.^65–67^ According to the crystal binding structure of succinyl-Lys peptide and SIRT5, researchers found that tyrosine 102 and arginine 105 are the special residues of SIRT5, in the deep end of substrate-binding pocket, which forms hydrogen bonds and ionic bonds with the carboxyl group of the succinyl lysine substrate. This might also suit for its binding with malonyl lysine substrate. Besides SIRT5, SIRT7 was also reported as a desuccinylase, which has close relationship with DNA damage.^68^ Whether SIRT7 could regulate the other two negatively charged acidic acyl groups is still unknown. The removal of S-form and R-form β-hydroxybutyryl, the polar acyl groups, is mediated by SIRT3 and HDAC1/HDAC2, respectively.^69,70^ The distinct backbone sensitivity of NAD^+^-dependent (SIRT3) and Zn^+^-dependent (HDAC1/HDAC2) subfamilies decides their chiral selectivity, which still needs in-depth study. Similar with Kbhb, HDAC1–3 and SIRT1–3 have been identified as delactylases in vitro. Besides, the de-L-lactylase activity of HDACs 1 and 3 have been verified in cells.^71^ The erasers of the hydrophobic crotonyl group are mainly class III HDACs, including SIRT1, 2, and 3.^72^

#### Erasers for protein myristoylation and palmitoylation

Depalmitoylation is carried out by serine hydrolases, including acyl-protein thioesterase (APT), palmitoyl protein thioesterase (PPT), and a family of mammalian α/β hydrolase domain-containing proteins (ABHDs). Erasers of lysine or N-terminal glycine myristoylation are totally different. The removal of lysine myristoylation is mainly mediated by SIRT6, which has a large hydrophobic pocket that makes it a perfect eraser for long-chain fatty acyl groups.^73^ N-terminal glycine myristoylation has long been regarded as an irreversible protein lipidation. However, in a recent study, researchers identified a new demyristoylase for human ARF1—invasion plasmid antigen J (IpaJ), a previously uncharacterized Shigella flexneri type III effector protein with cysteine protease activity.^74^

### Readers of protein acylation

Proteins containing either of the following five domains have been characterized as histone and nonhistone acylation readers and the bank is still being expanded.^4,75–77^ The first category are proteins with bromodomains (BDs), which include bromodomain and extra-terminal (BET) or non-BET family of proteins. The second are proteins with double PHD finger (DPF), including MOZ, MOF, and DPF2. The third are the YEATS family proteins, including AF9, YEATS2, GAS41, TAF14, Sas5, Yaf9, and ENL. The last two are proteins with double PH domain (such as Rtt106) and ZZ-type zinc finger domain (such as p300). Besides, many “writers” have dual identities as “readers” such as CBP (bromodomain), p300 (ZZ domain), MOF (DPF domain), and MORZ (DPF domain).

Studies on protein acylation readers are mostly focused on histone Kace, Kpro, Kcro, Ksuc, Kbhb, and nonhistone Kace. Few reports are on the readers of histone or nonhistone protein long-chain fatty acid lipidation, studies are still in need to explore whether there are unknown readers to bind such bulk acyl groups. One reader can recognize different types of protein acylation with its unique priority. For example, many readers of the YEATS type bind to K acylation through an aromatic cage, with the highest affinity towards crotonylation followed by the other acyl marks approximately in order of the length of the fatty acid chain.^78–80^ The readers usually translate the protein acylation marks to the signal in regulating protein transcriptional activity, DNA-binding ability, or degradation speed.

Among the readers, only the type of bromodomains (BRD4, BRD3, and PBRM1) have been reported to recognize acetylated nonhistone proteins, which are all transcription factors. BRD4 is a member of the BET family with two BDs (BD1 and BD2) reading acetylated RelA, ERG, Twist, and Snail. Both BD1 and BD2 domains of BRD4 could recognize and interact with RelA, recruiting CDK9 to phosphorylate the C-terminal domain of RNA polymerase II and facilitating the transcription of NF-κB-dependent genes.^81–83^ Similar with the working model of BRD4 on RelA, BRD4 binds with the ERG acetylated ^96^KGGK^99^ motif and the Twist diacetylated “histone H4-mimic” GK-X-GK motif to upregulate their transcriptional activity.^84^ Different from all the mechanisms mentioned above, BRD4 recognizes CBP-acetylated Snail (K146 and K187) to enhance its protein stability.^85^ BRD3 is another member of BET family with BD1 and BD2 domains. It has been reported to promote erythroid maturation through “reading” the acetylated GATA1 with its BD1 domain and promoting its stable association with chromatin.^86^ In summary, BRD4 and BRD3 use different BD domains (BD1, BD2, or both) to recognize the acetylated nonhistone proteins and regulate either their transcriptional activity or protein degradation, the selectivity of the recognition domains and the working mode is still being elucidated in the future. PBRM1 is the second most highly mutated tumor suppressor gene in kidney cancer with four BD domains. Recent studies found that PBRM1 reads acetylated K382 of p53 through its BD4 domain to promote the interaction of p53 with the promoter of its target gene p21.^87^ Whether there are any other readers of the bromodomain type or of other types that are responsible for the recognition of nonhistone protein acetylation and other kinds of acylation still needs to be studied.

## Protein acylation in human diseases

### Acetylation and its role in human diseases

Acetylation is a metabolic and chemical process, during which the acetyl group is attached to the protein/peptide or messenger RNA.^2,60^ In protein acetylation, the acetyl groups bind covalently to the lysine, serine or threonine residues of amino acids either in a nonenzymatic manner, especially in alkaline environments such as the mitochondrial matrix, or in an enzymatic manner.^88–91^

#### Protein acetylation in tumor

Protein acetylation and deacetylation mediated by KATs and HDACs can occur in either tumor cells or immune cells and eventually change the intrinsic tumor features or immune cell phenotypes. Hereafter, we summarize and discuss the effects of nonhistone protein acetylation or deacetylation in tumor development and progression that have been reported in the last 10 years.

##### Protein acetylation in the metabolic adaptation of tumors

**Fig. 2:**
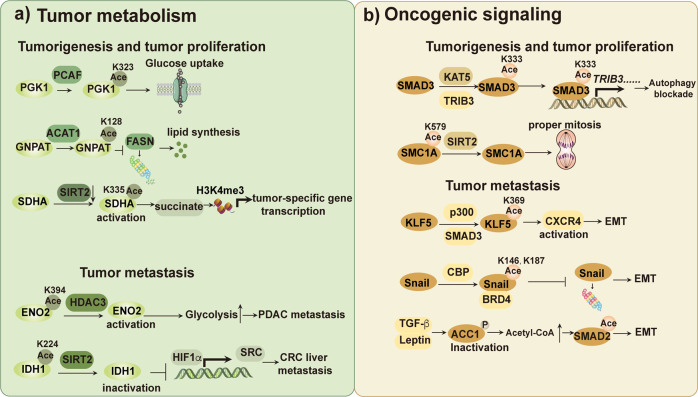
Protein acetylation in shaping tumor metabolism and oncogenic signaling. Protein acetylation usually influence tumor progression via regulating metabolic enzymes and oncoproteins. **a** Protein acetylation in the regulation of tumor metabolism. Metabolic enzymes responsible for tumorigenesis and proliferation are regulated by acetylation. PGK1 is acetylated by PCAF at K323 to promote glucose uptake. GNPAT is acetylated by ACAT1 at K128 to inhibit FASN degradation and enhance lipid synthesis. SIRT2 degradation leads to succinate production and H3K4me3 activation. The above effects either provide energy source for tumor proliferation or activate tumor-specific gene transcription. Besides, protein acetylation can also occur on metabolic enzymes responsible for tumor metastasis. ENO2 is deacetylated by HDAC3 at K394 to increase its activity and glycolysis. IDH1 involved in glutamine metabolism is deacetylated at K224 to inhibit its enzymatic activity and HIF1α-SRC transcription axis. Enhanced glycolysis and HIF1α-SRC transcription axis is closely connected with tumor metastasis. **b** Acetylation of oncogenic signaling proteins relates to tumorigenesis, proliferation and metastasis. TRIB3 promotes KAT5-mediated SMAD3 acetylation at K333 to promote the transcriptional activity of SMAD3, which positively regulates transcription of the downstream *TRIB3* and results in autophagy blockade. SIRT2 inhibits SMC1A acetylation at K579 to induce proper mitosis. SMAD3 recruits p300 to acetylate KLF5 at K369 and promote the expression of its target gene—*CXCR4* and EMT. BRD4 recognizes CBP-acetylated Snail (K146 and K187) to enhance its protein stability and promote EMT. ACC1 is phosphorylated and inactivated by leptin or TGF-β signaling, resulting in increased acetyl-CoA and SMAD2 acetylation, which finally upregulates SMAD2 transcriptional activity and EMT

Protein acetylation regulates tumor progression by regulating metabolic enzymes, affecting their enzymatic activity or stability (Fig. 2a). Vigorous glycolysis in tumors is closely related to acetylation. Phosphoglycerate kinase 1 (PGK1) and PGK2 are the only enzymes that produce ATP in the glycolysis pathway, in which PGK1 is overexpressed in liver cancer.^92,93^ Researchers found that PCAF-mediated K323 acetylation of PGK1 enhances its enzymatic activity and glucose uptake, elevating liver cancer cell proliferation, and tumorigenesis.^94^ Enolase 2 (ENO2) is another key glycolytic enzyme in the metabolic process of glycolysis and is overexpressed in prostate cancer, small-cell lung cancer, metastatic neuroblastoma, and leukemia.^95,96^ HDAC3-mediated deacetylation of ENO2 at K394 leads to its activation and enhancement of glycolysis, which finally results in the metastasis of pancreatic ductal adenocarcinoma (PDAC).^97^

Acetylation of enzymes in lipid metabolism have also been found to promote cancer development. Glyceronephosphate O-acyltransferase (GNPAT) is critical for the synthesis of fatty acids, which are dysregulated in hepatocellular carcinoma (HCC).^98^ The acetyltransferase ACAT1 is upregulated in response to extra palmitic acid (PA) and acetylates GNPAT at K128, which represses tripartite motif-containing 21 (TRIM21)-mediated ubiquitination and degradation of GNPAT. The accumulated GNPAT then represses the degradation of fatty acid synthase (FASN) mediated by TRIM21 and promotes fatty acid synthesis and hepatocarcinogenesis.^99^

Protein acetylation of the key enzymes in glutamine metabolism or in the TCA cycle is another good example in tumor. Isocitrate dehydrogenase 1 (IDH1) is involved in glutamine metabolism, which is located in the cytoplasm and converts isocitrate to α-KG. Researchers found that wild-type IDH1 is hyperacetylated at K224 in colorectal cancer (CRC), promoting CRC progression and liver metastasis. SIRT2 could deacetylate IDH1 at K224 and exhibit a tumor suppression function in a colon cancer cell model by inhibiting IDH1 enzymatic activity and the hypoxia-inducible factor 1α (HIF1α)-SRC transcription axis. The above examples indicate that the acetylation of metabolic enzymes play complicated regulatory role of enzymatic activity. Whether acetylation enhances or inhibits the enzymatic activity may depend on the spatial location of the acetylation site relative to the catalytic domain or degradation-related ubiquitination site.

##### Protein acetylation in oncogenic or tumor-suppressive signaling

In addition to metabolic enzymes, numerous oncogenic or tumor-suppressive signaling pathways are regulated by acetylation, involving in tumorigenesis, metastasis or drug resistance (Fig. 2b).

The pseudokinase tribbles homolog 3 (TRIB3) has been demonstrated to promote tumorigenesis in lung cancer, liver cancer, breast cancer, colorectal cancer and leukemia.^100–107^ Recently, our group elucidated that TRIB3 acts as an adaptor to recruit KAT5 to SMAD3, inducing phosphorylation-dependent SMAD3 K333 acetylation. This modification sustains SMAD3 transcriptional activity and subsequently enhances TRIB3 transcription, forming a positive feedback regulation loop. Metformin could inhibits KAT5-mediated SMAD3 K333 acetylation and TRIB3 expression, inhibiting the progression of melanoma.^108^ Protein acetylation also regulates mitotic catastrophe in cancer. Mitotic catastrophe can be defined as an oncosuppressive mechanism that eliminates mitosis-incompetent cells.^109,110^ SIRT2 is upregulated in early-stage carcinomas and deacetylates the structural maintenance of chromosome protein 1 (SMC1A), which then promotes its phosphorylation to properly drive mitosis. This permits tumor cells to escape mitotic catastrophe, thus allowing early precursor lesions to overcome oncogenic stress.^111^

Epithelial–mesenchymal transition (EMT) is a complex developmental program that enables cancer cells to acquire a more aggressive phenotype for metastasis and therapy resistance. Extensive crosstalk occurs between metabolism and EMT to coordinate tumor progression. The lipogenic enzyme acetyl-CoA carboxylase 1 (ACC1) was recently demonstrated to suppress breast cancer migration and invasion in a manner that was independent of fatty acid synthesis but was dependent on acetyl-CoA. Mechanistically, ACC1 is phosphorylated and inactivated by leptin or transforming growth factor-β (TGF-β) signaling, which is highly expressed in obesity. This results in increased acetyl-CoA and SMAD2 acetylation, which finally upregulates its transcriptional activity and promotes EMT programs in breast tumor cells.^112^ DOT1L is a histone methyltransferase that regulates various genes involved in cancer onset and metastasis.^113,114^ CBP promotes DOT1L acetylation at K358 and enhances its stability by preventing the binding of RNF8 and DOT1L. The stabilized DOT1L catalyzes the H3K79 methylation of genes involved in EMT, including Snail and ZEB1, thus promoting CRC metastasis.^115^ Krüppel-like Factor 5 (KLF5) is a key transcriptional factor in regulating cell proliferation, apoptosis, tumor cell stemness traits and EMT.^116,117^ Acetylation of KLF5 has been reported to play opposite roles in the progression of prostate cancer and breast cancer via regulating its transcriptional activity or protein stability. In prostate cancer (PCa), bone-borne TGF-β was found to promote the acetylation of KLF5, leading to osteoclastogenesis and chemoresistant bone metastatic formation. Mechanistically, SMAD3 recruits p300 to acetylate KLF5 at K369 and enhance its transcriptional activity, thus promoting the expression of its target gene—CXCR4. The increased CXCR4 then promotes IL-11 secretion and stimulates metastasis-associated SHH/IL-6 paracrine signaling.^118^ While, acetylation of KLF5 at the same lysine residue inhibit tumor progression in basal-like breast cancer (BLBC). Kong et al. found that HDAC inhibitors increase KLF5 acetylation at K369 to interrupt its association with BRCA1-associated protein 1 (BAP1) (a deubiquitinase), promoting its ubiquitination and degradation. This will decrease cell viability in BLBC cell lines.^119^ It is possible that there is a different secondary signal involved in the two types of tumors, leading to contradictory tumor biological outcomes, which deserves further investigation.

The most deeply studied tumor suppressor under acetylation regulation is p53. In response to cellular stresses, p53 transcriptionally participates in the modulation of multiple biological processes, including proliferation, cell cycle arrest, programmed cell death (apoptosis), cellular senescence, DNA repair, autophagy, oxidative response, and metabolic regulation.^120^ The acetylation of different lysine residues of p53 plays opposite roles in tumor progression.^121^ In response to DNA damage, p300/CBP mediates C-terminal (K370, K372, K373, K381, K382, and K386) and K101 acetylation to suppress p53-dependent apoptosis and ferroptosis.^122,123^ In addition, PCAF-mediated p53 K320 acetylation also promotes cell survival and inhibits apoptosis by selectively inducing the expression of antiapoptotic genes and repressing proapoptotic genes.^65^ In contrast, p300/CBP and MYST family (TIP60, MOF, and MOZ)-mediated acetylation of p53 at K120 and K164 inhibit tumor progression by promoting apoptosis. Cancer cell-intrinsic programmed cell death 1 (PD-1) has been suggested to suppress lung cancer progression. In a recent study, researchers demonstrated that PD-1 is a target gene of p53, and acetylation of p53 at K120 and K164 helps to promote PD-1 transcription and suppress lung cancer development in an immunity-independent manner.^124^

##### Tumor cell protein acetylation in shaping antitumor immune responses

Recently, protein acetylation in tumor cells or immune cells has been reported to shape the immunosuppressive tumor microenvironment by regulating immune cell exhaustion, activation, and infiltration.

**Fig. 3:**
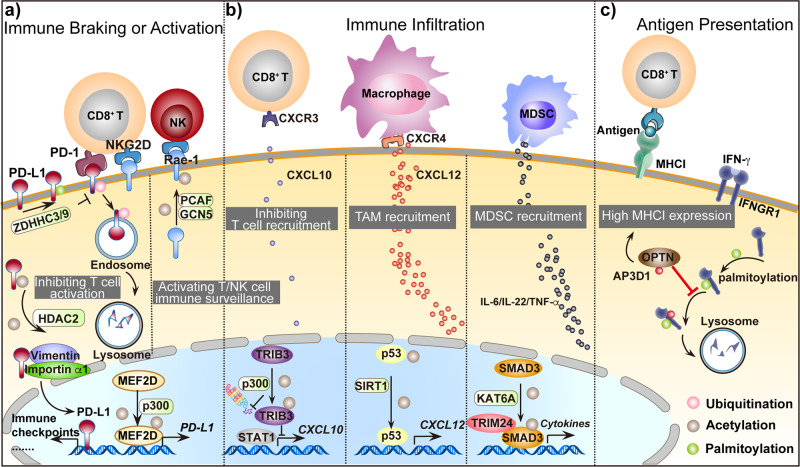
Protein acylation in shaping tumor immune microenvironment. Protein acylation helps to shape immunosuppressive tumor microenvironment via regulating immune cell activation, infiltration and antigen presentation. **a** Protein acylation in immune braking or activation. HDAC2 inhibits PD-L1 acetylation to increase its nuclear localization and immune checkpoints activation. P300 mediates MEF2D acetylation to promote PD-L1 transcription. ZDHHC3 and ZDHHC9 mediate PD-L1 palmitoylation to inhibit its lysosomal degradation. The three events will induce T cells exhaustion. Rae-1 is acetylated by PCAF and GCN5 to enhance its stability and activate NK/T cells killing ability. **b** Protein acylation in immune infiltration. P300-mediated TRIB3 acetylation inhibits T cells infiltration through inhibiting *CXCL10* transcription. SIRT1-mediated p53 deacetylation promotes TAM infiltration through secreting CXCL12. KAT6A-mediated SMAD3 acetylation results in its transactivation and the transcription of cytokines, including *IL-6/IL-12/TNF-α* and promotes MDSC infiltration. **c** Protein acylation in antigen presentation. OPTN interacts with AP3D1 to hinder its recognition of IFNGR1, thereby maintaining IFNGR1 stability and the integrity of downstream MHC-I signaling, promoting antigen presentation to T cells

Programmed cell death 1 ligand 1 (PD-L1), which is expressed on cancer cells, binds to the receptor PD-1 on T cells to prevent their proliferation and reduce the anti-tumor immune response, resulting in T-cell exhaustion.^125–127^ Researchers found that the acetylation of PD-L1 or related transcription factors could regulate its subcellular localization. Through interacting with vimentin and importin α1, PD-L1 shuttles from the plasma membrane into the nucleus, in which the deacetylation of PD-L1 by HDAC2 at K263 is a precondition.^128^ Nuclear PD-L1 then directly binds with DNA to regulate the transcription of multiple immune response-related genes and several immune checkpoints. Blocking PD-L1 nuclear translocation via HDAC2 inhibition enhances the PD-1 blockade therapy. Protein acetylation also affects PD-L1 transcription levels. Myocyte enhancer factor 2D (MEF2D) is a transcription factor that is overexpressed in HCC and is associated with poor survival of HCC patients.^129^ Researchers found that when HCC cells are exposed to interferon gamma (IFN-γ), p300 acetylates MEF2D and promotes its binding with the *PD-L1* promoter, leading to increased PD-L1 expression. Strategies to manipulate this pathway might increase the efficacy of immune therapies for HCC (Fig. 3a).^130,131^

In contrast to PD-L1, the natural killer group 2 member D (NKG2D) ligand Rae-1 binds with NKG2D in T cells or NK cells to activate their tumor-killing activity.^132^ Shedding, loss of expression, or internalization of ribonucleic acid export 1 (Rae-1) from the tumor cell surface leads to immune evasion, which is associated with poor prognosis in patients with cancer.^133–135^ Researchers found that GCN5 and PCAF acetylate Rae-1 at K80 and K87 to enhance its stability and protect Rae-1 from shedding to activate the immune surveillance of NK cells and CD8^+^ T cells. This observation may shed light on new targets for NKG2D immunotherapy in cancer treatment (Fig. 3a).^136^

Protein acetylation in tumor cells also influences immune cell infiltration by regulating the secretion of chemokines. SIRT1 was demonstrated to promote CXCL12 expression by inhibiting the acetylation of p53 in colorectal cancer. This induces tumor-associated macrophage (TAM) migration through the CXCL12/CXCR4 pathway. The recruited TAMs further inhibit the proliferation and activity of CD8^+^ T cells, resulting in CRC progression.^137^ In some cases, the acetylation of oncoproteins could regulate both cancer stemness features and immune cell recruitment at the same time. Our recent study revealed that p300 induces TRIB3-K240 acetylation under high-glucose conditions. This modification attenuates the association of TRIB3 with the E3 ligase SIAH1 and inhibits its ubiquitination and degradation.^138^ On one hand, aberrant highly expressed TRIB3 enhances the stemness of cancer cell by activating the Wnt/β-catenin signaling pathway.^102^ On the other hand, TRIB3 represses the STAT1-CXCL10 axis and reduces CD8^+^ T-cell infiltration, resulting in the immune evasion of CRCs. This work highlighted a potential therapeutic target for treating immunologically “cold” CRCs.^101^ The acetylation of SMAD3 is another good example. SMAD3 often acts as a mediator of TGF-β superfamily that modulates signaling and has been implicated as a driving event in cancer metastasis.^81^ The acetylation of SMAD3 at K20 and K117 by KAT6A promotes its association with oncogenic chromatin modifier TRIM24 and disrupts its interaction with the tumor suppressor TRIM33, enhancing the transcription of immune response-related cytokines. This enhances myeloid-derived suppressor cell (MDSC) recruitment and breast cancer stem-like cell stemness, which promote triple-negative breast cancer (TNBC) metastasis (Fig. 3b).^139^

##### Immune cell protein acetylation in shaping of antitumor immune responses

Protein acetylation in immune cells also regulates tumor-killing activity. STAT6 acetylation in TAMs affects their polarization and tumor-killing ability. STAT6 is known to drive macrophage M2 polarization. The K383 of STAT6 is acetylated by the acetyltransferase CBP during macrophage activation to suppress macrophage M2 polarization. Mechanistically, TRIM24, a CBP-associated E3 ligase, promotes STAT6 acetylation by catalyzing CBP ubiquitination at K119 to facilitate the recruitment of CBP to STAT6. In contrast, STAT6 mediates the suppression of TRIM24 expression in M2 macrophages, contributing to the induction of an immunosuppressive tumor niche.^59,140^

#### Protein acetylation in Alzheimer’s disease

Alzheimer’s disease (AD) is the most common neurodegenerative disease, which is pathologically caused by neurofibrillary tangles (NFTs) and extracellular accumulation of beta-amyloid (Aβ). In AD, highly phosphorylation and aggregation of tau protein contribute to the formation of NFTs and to mediate Aβ toxicity. Therefore, reducing tau is a prospective strategy for AD therapy. Tau is majorly degraded by macroautophagy and chaperone-mediated autophagy (CMA) to avoid its excessive accumulation in AD.^141^ Acetylation of soluble tau is an early pathological event in neurodegeneration. Numerous studies have reported the complex relationship between tau acetylation and its protein stability. Acetylation of tau at K174 or K274 impedes CMA, which alters tau homeostasis and contributes to aggravate disease progression.^142^ Another group demonstrated that the loss-of-function mutation in the TSC Complex Subunit 1 (*TSC1)* gene results in upregulated p300 and damaged SIRT1 enzymatic activity, leading to tau acetylation and preventing tau clearance via CMA.^143^ HDAC6 was reported to deacetylate and inhibit hyperphosphorylation of tau, which alleviates neurodegeneration and cognitive decline.^144^ While another group has discovered the opposite relationship between tau acetylation and its protein stability. Choil et al. found that acetylation of tau at K274, K290, K321, and K353 recruits chaperone proteins, including Hsp40, Hsp70, and Hsp110, which facilitates novel tau E3 ligases binding and tau degradation.^145^ The contradictory phenomena might due to the different acetylated lysine residues in tau, which indicate the complicated regulatory network of tau PTMs.

#### Protein acetylation in hepatic steatosis

The liver is the metabolic hub of glucose, fatty acid, and amino acid, which is largely affected by the metabolic enzymes and associated PTMs. Protein acetylation has been linked with both alcoholic (AFLD) and non-alcoholic fatty liver disease (NAFLD) through regulating transcription factors or metabolic enzymes. SIRT2 has been demonstrated to play opposite roles in AFLD and NAFLD via regulating different transcription factors. SIRT2 mediates CCAAT/enhancer-binding protein beta (C/EBPβ) deacetylation at K102 and K211 to inhibit its ubiquitination and degradation.^146^ The accumulated C/EBPβ promotes ethanol-induced liver injury via regulating the expression of target genes involved in adipogenesis, gluconeogenic pathway, liver regeneration, and so on. While, in the NAFLD, SIRT2 prevents liver steatosis and metabolic disorders by deacetylating and stabilizing hepatocyte nuclear factor 4α (HNF4α) at K458.^147^ In both of the two studies, Kace works on the transcription factors via regulating their protein stability but not transcriptional activity, providing a novel regulatory mechanism of acetylation on transcription factors. Besides, it’s because of the different functions of the substrates that make similar stability regulation by SIRT2-mediated deacetylation with opposite effects on disease progression. Except for the transcriptional factors, the acetylation of key enzymes in glucose metabolism—lactate dehydrogenase-B (LDHB) also regulates NAFLD. PCAF-mediated acetylation of LDHB at K82 was found to significantly decrease its enzymatic activity and impair hepatic lactate clearance, resulting in lactate accumulation, which exacerbates lipid deposition and inflammatory responses by activating histone hyperacetylation in high-fat diet (HFD)-induced NAFLD.^148^ All of the evidence suggests the importance and complex of Kace in regulating metabolic diseases and provides potential therapeutic targets from the view of protein PTMs.

#### Protein acetylation in immune and infectious disease

Protein acetylation has close relationship with immune response in chronic inflammation and virus infection. In the aging-associated chronic inflammation, the acetylation of the inflammasome NLRP3 in macrophages is critical to its assembly and activation, leading to the production of inflammatory cytokines IL-1β and IL-18.^149^ In the innate immune response to DNA virus, the DNA sensor cGMP-AMP synthase (cGAS) senses cytosolic microbial or self DNA to initiate a MITA/STING-dependent innate immune response. Acetylation of cGAS by KAT5 in its N-terminal domain promotes its DNA-binding ability, increasing the transcription of downstream antiviral genes.^150^ In addition to the DNA virus infection, acetylation of OTU deubiquitinase 3 (OTUD3) has been verified to be critical to the RNA virus infection.^151^ Since 2019, SARS-CoV-2 has caused an ongoing pandemic of coronavirus disease 2019 (COVID-19) worldwide.^152^ Protein acetylation has also been studied in this severe acute respiratory syndrome. Acetylated K676 of TGF-β-induced protein (TGFBIp) was consistently elevated in the blood of patients with SARS-CoV-2 pneumonia, especially in patients of the intensive care unit (ICU) compared to non-ICU patients, suggesting it as a severity diagnostic biomarker.^153^ Although the mechanisms are still unclear, it can be predicted that protein Kace might be a potential prognostic or therapeutic target of the immune or infectious diseases.

### Succinylation and its role in human diseases

Succinylation has mainly occurred in the mitochondria which was identified and verified by Zhang et al.^6^ in 2011 by mass spectrometry and protein sequence alignment in vivo.^85,154^ Different from acetylation, Ksuc causes greater changes on lysine residues in charge (from +1 to −1) and structure, which is likely to have a significant impact on the substrate proteins. In detail, the positively charged chains of lysine residues in physiological pH plays critical roles in protein folding and the formation of noncovalent interactions such as the leucine zipper, as well as in general acid-base catalyzed enzymatic reactions in which proton transfer is required.^155^ Therefore, Ksuc is likely to lead to significant changes in PPI, enzymatic or transcriptional activities. Studies have discovered that most of the succinylated proteins are involved in the regulation of energy metabolism and translation, and nearly every enzyme of the TCA cycle is succinylated, implying a possible role for Ksuc in energy metabolism.^156^ Nonhistone protein Ksuc has been studied in tumor, COVID-19, metabolic diseases, neurological disease, cardiovascular disease, immune system diseases, and mitochondrial diseases.^157^

#### Protein succinylation in tumor

Nonhistone Ksuc mainly regulate tumor development through influencing metabolic reprogramming, oncogenic signaling pathways and suppressive immune microenvironment.

##### Protein succinylation in the regulation of tumor anaerobic glycolysis and serine metabolism

**Fig. 4:**
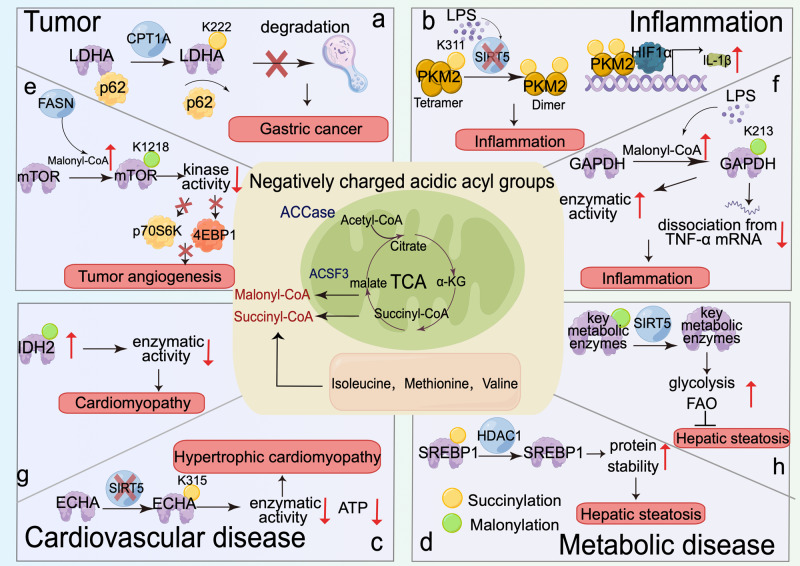
Protein succinylation and malonylation on metabolic enzymes or kinases in tumor, inflammatory, cardiovascular and metabolic diseases. The negatively charged acidic acyl groups including malonyl group derived from acetyl-CoA or malate, and succinyl group derived from α-KG or amino acids take part in the PTMs of metabolic enzymes in numerous kinds of diseases. **a**, **e** Protein Ksuc and Kmal in tumor. CPT1A mediated LDHA succinylation at K222 to inhibit its autophagic degradation via p62 to accelerate gastric cancer (**a**). Depletion or inhibition of FASN enhances malonyl-CoA level and promotes mTOR malonylation at K1218 to downregulate its kinase activity and the subsequent phosphorylation of p70S6K/4EBP1, promoting endothelial cells proliferation and tumor angiogenesis (**e**). **b**, **f** Protein succinylation and malonylation in metabolic enzymes play critical roles in LPS-induced inflammation of macrophages. LPS inhibits SIRT5 mediated desuccinylation of PKM2 at K311 to inhibit its kinase activity and increase its nuclear translocation by promoting PKM2 tetramer-to-dimer transition. The nucleus PKM2 interacts with HIF1α to promote IL-1β transcription and inflammation (**b**). LPS stimulation enhances malonyl-CoA level and promotes GAPDH malonylation at K213 in macrophages, leading to its increased enzymatic activity and dissociation from TNFα mRNA, promoting TNFα expression and inflammation (**f**). **c**, **g** Protein succinylation and malonylation in metabolic enzymes play critical roles in cardiovascular disease. Knockout of SIRT5 results in increased Ksuc in ECHA at K315, inhibiting its enzymatic activity and ATP production and promoting hypertrophic cardiomyopathy (**c**). IDH2 malonylation decreases its enzymatic activity and promotes cardiomyopathy (**g**). **d**, **h** Protein succinylation and malonylation in metabolic enzymes play critical roles in metabolic disease. HDAC1 inhibits SREBP1 succinylation and increases its protein stability, promoting hepatic steatosis (**d**). SIRT5 mediates several key metabolic enzymes malonylation to promote glycolysis and FAO, inhibiting hepatic steatosis (**h**)

LDHA mediates the last step in the anaerobic oxidation of glucose and catalyzes the formation of lactate. Protein succinylation mediated by CPT1A has been demonstrated to promote the proliferation and invasion of gastric cancer (GC) by regulating LDHA succinylation. Mechanistically, CPT1A succinylates LDHA at K222, which thereby reduces the binding of K63-ubiquitinated LDHA with SQSTM1 and inhibits its autophagic degradation. Overexpression of a succinylation-mimic mutant of LDHA could promote tumor cell proliferation, migration and invasion (Fig. 4a).^158^ SIRT5 is both a deacetylase and desuccinylase. In CRC and osteosarcoma, SIRT5 desuccinylates mitochondrial serine hydroxymethyltransferase (SHMT2) at K280 to increase its enzymatic activity, driving serine catabolism and promoting tumor proliferation.^159^ These results suggested that protein succinylation is used by cancer cells to adapt to metabolic status for rapid growth.

##### Protein succinylation and desuccinylation in the regulation of oncogenic signaling

S100 proteins are a family of calcium‐binding cytosolic proteins that possess a wide range of intracellular and extracellular functions and play pivotal roles in tumor migration and invasion. CPT1A-induced S100A10 succinylation at K47 suppresses its ubiquitylation and subsequent proteasomal degradation. Succinylated S100A10 is highly expressed in GC and promotes GC invasion.^160^ Notably, protein succinylation also demonstrates tumor-suppressive effect in the context of cancer types. Using proteomic technologies, lower lysine succinylation was found in esophageal squamous cell carcinoma (ESCC) than in nonmalignant controls. The decreased succinylation may be attributed to the decreased succinyl-CoA, leading to abnormal metabolism in ESCC. In addition, functional assays through either chemical or genetic approaches demonstrated that hyposuccinylation elevates the migratory ability of ESCC cells both in vitro and in vivo. Using tumor tissue samples from ESCC patients, the authors confirmed that the degree of lysine succinylation was higher in adjacent nonmalignant esophageal epithelium than in cancer tissues. These observations highlight the complicated effect of succinylation modification in cancer biology.^161^

##### Protein succinylation in shaping the immunosuppressive tumor microenvironment

Succinylation regulators have an intense relationship with immune cell phenotypes, especially regulatory T cells (Tregs). To systematically study the role of succinylation regulators in tumors, Lu et al. performed a comprehensive pan-cancer analysis on four well-known succinylation regulators (CPT1A, KAT2A, SIRT5, and SIRT7). They found that KAT2A was prominently upregulated in all types of tumors compared to the corresponding normal tissues. SIRT7 was significantly upregulated in nine of ten tumor types and downregulated in colon adenocarcinoma. The expression of CPT1A and SIRT5 showed high heterogeneity in different tumors, which is consistent with the previously reported studies. The researchers performed further analysis on clear cell renal cell carcinoma (ccRCC) since all four regulators are associated with the overall survival of patients with ccRCC. Using analytical bioinformatics, they found that SIRT7-high, SIRT5-low or CPT1A-low might contribute to the infiltration of forkhead box P3^+^ (FOXP3^+^) Tregs, which induce an immunosuppressive microenvironment. As SIRT5 and SIRT7 are both regulators of deacetylation and desuccinylation, the regulation of Treg cell infiltration might be related to the changes in these two post-translational modifications.^162^

#### Protein succinylation in inflammatory and infectious disease

The internal inflammatory reaction is crucial in killing invaders and avoiding harmful infection. However, if the inflammatory response is out of control or persists for a long time, it will cause diseases such as rheumatoid arthritis, inflammatory bowel diseases and potentially fatal sepsis. Lipopolysaccharide (LPS) strongly increases the intermediate succinate level in TCA cycle of macrophages, which stabilizes HIF1α to induce IL-1β expression, establishing the relationship of succinate and Ksuc with inflammation.^163^ Mechanistically, LPS-activated macrophages undergo a metabolic shift from dependence on mitochondria-produced ATP to reliance on aerobic glycolysis, where pyruvate kinase M2 (PKM2) is a critical determinant.^164,165^ They found that LPS results in a reduced desuccinylation activity of SIRT5. This will in turn increase PKM2 Ksuc at K311 to inhibit its kinase activity and increase its nuclear translocation by promoting PKM2 tetramer-to-dimer transition, which leads to the interaction of PKM2 with HIF1α and promotes IL-1β transcription and DSS-induced colitis in mice.^166^ The studies above suggest a key role of protein PTM in linking metabolic reprogramming and inflammation (Fig. 4b).

Novel antiviral therapies against SARS-CoV-2 and the emerging variants are still in urgent need. Viruses can’t replicate by themselves, hence they have evolved to alter the host cellular pathways and protein PTMs to meet their requirement for replication. After SARS-CoV-2 infection, dramatic upregulation of protein Ksuc in host (Caco-2 cells) was observed by using a quantitative mass spectrometry-based succinylproteomic strategy.^167^ Most of the hypersuccinylated proteins are enzymes in the TCA cycle, including citrate synthase (CS), aconitase 2 (ACO2), dihydrolipoamide S-succinyltransferase (DLST), succinate-CoA ligase GDP/ADP-forming subunit alpha (SUCLG1), succinate-CoA ligase ADP-forming subunit beta (SUCLA2), succinate dehydrogenase complex flavoprotein subunit A (SDHA), fumarate hydratase (FH), oxoglutarate dehydrogenase (OGDH), and malate dehydrogenase 2 (MDH2), leading to the inhibition of cellular metabolic pathways. Besides, viral nonstructure protein NSP14 is capable to participate in succinylation through interacting with host SIRT5. Indeed, SIRT5 activators and CPT1A inhibitors have been confirmed with potential antivirus effects. This study first provides evidence for the close relationship between metabolism and virus infection from the view of PTMs, which opens a new window for exploring anti-COVID-19 strategy in the future.

#### Protein succinylation in cardiovascular disease

Heart is the organ with huge energy supply need, which depends on healthy TCA cycle and oxidative phosphorylation. Researchers found that heart has the highest concentration of succinyl-CoA which has close relationship with TCA cycle. Therefore, they began to explore the function and molecular mechanism of Ksuc in ischemic cardiomyopathy or hypertrophic cardiomyopathy. In one study, Sadhukhan et al. found that Sirt5^−/−^ mice develops hypertrophic cardiomyopathy with hyper protein succinylation in the heart. ECHA, a protein involved in fatty acid oxidation, was identified with the most succinylation sites (at 28 lysine residues). In detail, depletion of SIRT5 enhances ECHA Ksuc at K315, which inhibits ECHA enzymatic activity, leading to defective fatty acid metabolism, decreased ATP production, and hypertrophic cardiomyopathy (Fig. 4c).^40^ In another study, differential protein expression and succinylated lysine residues in myofibrils from failing ischemic cardiomyopathy hearts and non-failing hearts in patients were evaluated. Increased succinyl-CoA synthetase activity and succinyl-CoA turnover were found in ischemic heart failure samples, which results in significant decrease in protein Ksuc.^168^ From the evidence, one can conclude that protein hypersuccinylaiton promotes hypertrophic cardiomyopathy, while there is a decrease of protein succinylation in the ischemic failure heart. The seemingly contradictory results might because: (1) Heart failure is the end of hypertrophic cardiomyopathy, the decreased protein Ksuc might be a compensatory performance. (2) There might be species differences between mice and human. Collectively, the relationship between succinylation and cardiovascular disease is still worthy to be deeply studied.

#### Protein succinylation in metabolic diseases

LC–MS/MS analysis have suggested a tight connection between succinylome and diabetes/hepatic steatosis.^169,170^ Ksuc of optineurin (OPTN), the autophagy receptor, and sterol-regulatory element binding protein 1c (SREBP1c), the key transcription factor in de novo lipogenesis, have been studied in diabetic retinopathy (DR) and hepatic steatosis respectively in depth. DR is a neural and microvascular complication of diabetes and remains the leading cause of blindness in working-aged people (Fig. 4d).^171^ Elevated Ksuc level of OPTN at K108 with an unchanged protein stability was found in a treptozotocin (STZ)-induced type 1 diabetes rat model, which results in autophagic flux blockade and accumulation of oxidatively damaged proteins or organelles in the cytoplasm under high-glucose conditions. It is possible to speculate that the added large acidic succinyl group inhibits the function of OPTN as a receptor by affecting PPI. In the study of hepatic steatosis, Guo et al. found that the subunit of nuclear factor-κb (NF-κB)—p50 stabilizes HDAC1 to downregulate SREBP1c Ksuc, which increases SREBP1c stability and aggravate hepatic steatosis.^172^ The different influence of Ksuc on protein stability in S100A10 and SREBP1c indicate an uncovered complex regulatory mechanism of Ksuc.

#### Protein succinylation in AD

AD has a close relationship with abnormal brain glucose metabolism and accumulation of plaques and tangles. Nonhistone Ksuc has been found to be closely connected with pathological characteristics of AD. In a TMT labeled MS2-based quantitative proteomic study, researchers found that succinylation of multiple mitochondrial proteins declined, and succinylation of small number of cytosolic proteins increased.^173^ The largest increases occurred at critical sites of amyloid precursor protein (APP) (K612) and microtubule-associated tau (K311), which are crucial to AD progression.^174–177^ Mechanistically, succinylation of APP disrupted its normal proteolytic processing thereby promoting Aβ accumulation and plaque formation. Meanwhile, succinylation of tau promoted its aggregation to tangles and impaired microtubule assembly. This study suggested that targeting succinylation of tau, a star AD drug target, may have therapeutic potential against AD in the future.

### Malonylation and its role in human diseases

Protein Kmal and its deacylase SIRT5 was identified by the group of Zhao in 2011 after the discovery of Ksuc through mass spectrometry and protein sequence-database searching.^178^ They identified 25 peptides in 17 nonhistone proteins in HeLa cells, most of which are metabolic enzymes, including glyceraldehyde-3-phosphate dehydrogenase (GAPDH), NOP2/Sun RNA methyltransferase 2 (NSUN2), PGK1, ENO1, MDH2 and aldolase, fructose-bisphosphate A (ALDOA). As described above, malonyl-CoA is either derived from acetyl-CoA catalyzed by ACCase or malonate catalyzed by acyl-CoA synthetase family member 3 (ACSF3),^179–182^ which has a close relationship with glucose and fatty acid metabolism. Besides, malonyl-CoA has been shown to elevate in the skeletal muscle and the liver of OLETF rats and in muscle biopsies of patients with obesity and type 2 diabetes (T2D).^183^ The malonylation proteome reveals that Kmal and its regulation by SIRT5 were more prevalent in the cytosol than mitochondria, which is opposite to Ksuc with a major distribution in mitochondria.^184^ Nonhistone protein Kmal has been demonstrated to participate in metabolic-associated diseases, including metabolic syndrome, cardiovascular disease, tumor, and non-metabolic diseases, including inflammation.^185^ In detail, Kmal can be involved in the regulation of protein–protein interaction, mRNA binding ability, and enzymatic activity via changing protein charge from +1 to −1.

#### Protein malonylation in tumor

Protein Kmal has been suggested to promote cancer via increasing angiogenesis and F-actin nuclear polymerization. Bruning et al. has built the link between fatty acid synthesis with tumor angiogenesis via mTOR malonylation.^186^ They demonstrated that knockdown or inhibition of FASN helps interrupting the transformation of malonyl-CoA to palmitate, increasing malonyl-CoA accumulation and mTOR K1218 malonylation. This will in turn downregulate mTOR kinase activity and subsequent phosphorylation of p70S6K/4EBP1, promoting endothelial cells proliferation (Fig. 4e). Metastasis is the most common reason for HCC treatment failure and F-actin structure is known to link with the invasive and metastatic phenotypes of cancer cells.^187,188^ Recently, Kmal was found to promote lung metastasis of liver cancer via promoting polymerization of nuclear actin.^189^ In this study, the authors focused on the major actin-binding protein mouse diaphanous 2 (mDia2), which contains nuclear localization sequence (NLS) that contributes a lot to actin nuclear translocation and polymerization. They found that loss of mitochondrial transcription factor A (TFAM), a pivotal regulator of mitochondrial biogenesis, will block TCA cycle and cause the accumulation of cytoplasmic acetyl-CoA and its derivative malonyl-CoA, promoting mDia2 Kmal. Mechanistically, malonylation of NLS in mDia2 facilitates its interaction with importin α1 and subsequent nuclear translocation together with actin. Consequently, polymerization of nuclear actin allows chromatin compaction to be reordered to access genetic loci for transcription or repair.^190^ In this study, malonylation acts as a sensor to build a bridge between mitochondrial biogenesis and metastasis. It is possible that the influence on PPI of Kmal is due to the changed electrostatic interactions and protein conformation caused by the protein charge state switch.

#### Protein malonyaltion in inflammation

Pro-inflammatory macrophages, such as those activated by LPS, are highly glycolytic with a disrupted TCA cycle.^191^ In a recent study, Galván-Peña et al. found that malonylation of a wide variety of proteins occurs in macrophages in response to LPS.^192,193^ They focused on GAPDH, a critical enzyme in glycolysis, and creatively connect metabolic reprogramming with innate immune response via malonylation. This is the first time that protein Kmal was found in immune cells. In detail, LPS upregulates the catalytic activity of ACC1 to provide more malony-CoA for GAPDH Kmal at K213, leading to the increment of its enzymatic activity and dissociation from TNFα mRNA to promote TNFα translation (Fig. 4f). This study is likely to expand our understanding of underlying processes in infection and inflammation from the view of metabolism and protein acylation, and potentially indicate new therapeutic strategies to limit inflammation in disease.

#### Protein malonylation in cardiovascular disease

Malonylation of proteins especially IDH2, a key enzyme in the TCA cycle and mitochondria, has been explored in cardiomyopathy. Peoples et al. have developed the cardiomyopathy model by using mice with cardiomyocyte-specific depletion of mitochondrial phosphate carrier (SLC25A3), which causes defective mitochondrial ATP synthesis.^194^ They discovered a striking pattern of acylome remodeling with significantly increased acetylation and malonylation of mitochondria proteins. IDH2 is observed with both upregulated Kace and Kmal, as well as enhanced enzymatic activity. Kace of IDH2 enhances its enzyme activity through neutralizing the positive charge of target lysine, while Kmal plays an opposite role via imparting a negative charge. Deep study illuminated that the increased IDH2 activity is the competing results of its Kace versus Kmal. The biological function of the observed hypermalonylation in cardiomyopathy is still unclear. How does the different protein acylations coexist and compete with each other is worthy to be studied. In another study of cardiac hypertrophy, lower protein malonylation was observed in both transverse aortic constriction (TAC) induced animal model and angiotensin II induced cell model. Among the identified 330 proteins with Kmal, IDH2 was also found to show a significant decrease of Kmal with enhanced enzymatic activity in cardiac hypertrophy (Fig. 4g).^195^ In the two studies above, it is consistent that upregulation of IDH2 activity in cardiomyopathy connects with disease progression. Whereas IDH2 malonylation is completely opposite, reflecting delicate changes of malonylation in response to genetic, mechanical or pharmacological stresses.

#### Protein malonylation in metabolic disease

SIRT5 is a NAD^+^-dependent lysine deacylase responsible for removing malonyl groups. Using affinity enrichment and label free quantitative proteomics, researchers had characterized the SIRT5-regulated lysine malonylome in wild-type (WT) and Sirt5^−/−^ mice. They concluded that SIRT5 regulates both cytosolic and mitochondrial protein malonylation with glycolysis as a major target, linking Kmal with glucose metabolism.^58^ Based on their research, another group constructed the hepatic Sirt5-overexpressing ob/ob mouse model to study the biological role of SIRT5 and malonylation in pathological state of obesity.^196^ They demonstrated that overexpression of Sirt5 results in decreased malonylation and succinylation in hepatic cells, improved cellular glycolysis, suppressed gluconeogenesis, enhanced fatty acid oxidation, and attenuated hepatic steatosis. It is possible that high levels of sirtuins improve the activity of metabolic pathways and reverse some abnormal phenotypes probably by lowering the modification of key metabolic enzymes (Fig. 4h). This study provides an alternative view to understand the mechanism of metabolic abnormality in obesity and T2D, which would benefit for finding novel treatment strategy against these diseases.

### Protein crotonylation and its role in human diseases

Kcro has a conjugated double bond that differs from other protein acylation modifications.^197^ It was first identified in 2011 as an evolutionarily conserved modification in histone proteins via integrated, mass spectrometry-based proteomics approach.^197^ Six years later, two studies on the identification of nonhistone protein crotonylation have been developed by using the pan lysine crotonylation (α-Kcro) antibody and LC–MS/MS. In one study, 2696 lysine crotonylation sites were identified on 1024 proteins in the human lung adenocarcinoma cell line H1299, in which 40% of crotonylated proteins were in the cytoplasm, 27% were in the nucleus and 13% were in the mitochondria.^198^ In another study, 453 crotonylated proteins were identified from HeLa cells, in which 62.3% of the proteins were distributed in the nucleus, 9.4% were both distributed in nuclear and cytoplasmic localization, and 28.3% in other cellular components.^199^ These studies have expanded our understanding of this modification in regulating nonhistone proteins. Besides, it’s possible to speculate that different cell types and disease conditions might accurately decide the different Kcro subcellular distributions. Recently, novel bioinformatics or computational tools for accurate and fast identification of Kcro sites on human nonhistone proteins have been developed, which may serve as an efficient means to assist academicians with their experimental researches.^200,201^ Histone and nonhistone Kcro have been demonstrated to participate in tumor and ischemic heart disease (IHD) via regulating metabolic enzymatic activity or protein stability.^202,203^

#### Protein crotonylation in tumor

In recent years, lysine crotonylation was demonstrated to play opposite roles in diverse cancer types by regulating metabolic enzymes or oncogenic proteins. It is downregulated in liver, stomach, and kidney carcinomas and upregulated in thyroid, esophageal, colon, pancreatic, and lung carcinomas.^204^ In liver cancer, upregulating the crotonylation level by knocking down HDACs or adding HDAC inhibitors could inhibit hepatoma cell motility and proliferation. However, it helps to promote tumor progression in CRC and cervical carcinoma.

##### Protein crotonylation regulates glycolysis to aggravate tumor progression

ENO1 is an enzyme in glycolysis that catalyzes glyceric acid-2-phosphate to phosphoenolpyruvate. In addition to its glycolytic function, there is growing evidence to show that ENO1 is an oncogene in CRC tissues.^205,206^ It was reported that CBP mediates the crotonylation of ENO1 at K420 to promote tumor growth, migration, and invasion of CRC cells in vitro by enhancing ENO1 enzymatic activity and regulating the expression of tumor-associated genes.^207^

##### Protein crotonylation in the regulation of oncogenic signaling

Heterogeneous nuclear ribonucleoprotein A1 (HNRNPA1) is highly expressed in a variety of cancers and closely associated with cancer initiation and progression.^208–210^ P300-mediated lysine crotonylation of HNRNPA1 helps to upregulate its protein expression, which in turn promotes HeLa cell proliferation, invasion, and migration.^211^ However, whether crotonylation hampered HNRNPA1 ubiquitination and degradation to enhance its protein stability still needs to be investigated.

#### Protein crotonylation in cardiovascular disease

Myocardial ischemia-reperfusion injury contributes to adverse cardiovascular outcomes after myocardial ischemia or cardiac surgery. A proteomic analysis revealed that cardiac ischemia-reperfusion injury causes Kcro of proteins associated with cardiomyocyte contractility, resulting in the disruption of cardiomyocyte mitochondrial, sarcomere architecture, and gap junction, as well as the induction of cardiomyocyte apoptosis.^203^ The pathological role of Kcro modification on cytoskeletal protein tropomyosin alpha-1 chain (TPM1) and metabolic enzyme IDH3a were further investigated. In detail, Kcro mimicking mutants of IDH3a (K199Q) and TPM1 (K28/29Q) not only protect cardiomyocyte from apoptosis by inhibiting Bcl-2 adenovirus E18 19-kDa-interacting protein 3 (BNIP3)-mediated mitophagy or cytoskeleton structure rearrangement but also preserves post-injury myocardial function by inhibiting fibrosis and apoptosis. However, how does the hydrophobic crotonyl group regulate the biological function of IDH3a and TPM1 is still worth to be studied.

### β-hydroxybutyrylation and its role in human diseases

Ketone bodies, including β-OHB, acetoacetate, and acetone, are intermediate metabolites during fatty acid oxidation.^212^ In the year of 2016, Xie et al. identified a new type of histone modification—Kbhb. They demonstrated that Kbhb is significantly induced during prolonged fasting in mouse liver and is associated with genes upregulated in starvation-responsive metabolic pathways.^9^ Koronowski et al. also found that dietary and disease states of ketogenesis evoke Kbhb across the cellular proteome.^42^ Kbhb occurring on histone proteins plays important roles in liver cancer and major depressive disorder.^213,214^ Studies on nonhistone proteins’ Kbhb are limited and still ongoing.

#### Protein β-hydroxybutyrylation in tumor

In a study of tumor and diet, researchers found that β-OHB produced by ketogenic diet with low carbohydrate and high fat inhibits CRC development.^215^ On the contrary, β-OHB treatment induced Kbhb of p53 at K120, K319, and K370 by CBP/p300 to attenuate the cell growth arrest and apoptosis-inducing functions of p53.^216^ Mechanistically, p53 kbhb results in reduced p53 acetylation and inhibits the expression of *p21* and *PUMA*, two downstream target genes of p53. In fact, ketone bodies, such as β-OHB, are reported to play double-sword roles in cancer biology.^217,218^ Attenuation of p53 activity upon β-OHB-induced Kbhb might partially explain the complex role of ketone bodies in cancer. The inconsistency of these studies may be because that p53 mutation has not been taken into consideration. The above evidence also reminds us that specific diet intervention or metabolite supplementation should be carefully considered, especially for cancer patients.

### Lactylation and its role in human diseases

Studies over the last few years have documented that lactate is not only the end product of glycolysis, but also the major circulating energy source and the regulator of multifunctional signaling molecules in human diseases, especially tumors and inflammation.^219,220^ For example, lactate has been reported to promote tumor progression by helping cancer cells resist the stress of glucose deprivation, reducing immune surveillance of CD8^+^ T cells or NK cells, and creating an acidic tumor microenvironment that benefits tumor metastasis.^221–224^ Besides, Synovial lactate is a promising biomarker to distinguish septic from aseptic arthritis.^225^ However, the underlying mechanism is still unclear. In 2019, lactate-derived protein Klac was identified as a novel acylation happened on histone proteins by Zhang et al.^12^ They demonstrated that the abundance of Klac is mainly determined by the concentration of lactate and the activity of glycolysis, providing a novel possible working mode of lactate. Klac of histone proteins has been associated with lung myofibroblasts, AD and tumor including CCRC, liver cancer, and non-small-cell lung cancer (NSCLC).^226–231^

With the progress of analytical chemistry, numerous nonhistone Klac have been identified, expanding beyond its transcriptional regulation function. Utilizing the signature cyclic immonium (CycIm) ion of Klac generated from the linear immonium (LinIm) ion during MS/MS, Wan et al. has developed a straightforward approach to enable the confident identification of Klac by LC–MS/MS. They unveiled a widespread lactylation proteome beyond histones in both cytoplasm and nucleus from not only the enriched lactylproteome but also the existing unenriched human proteome resources.^232^ With the help of other chemical tools such as alkynyl-functionalized bioorthogonal chemical reporter (YnLac), nonhistone Klac has also been reported by other studies in recent years.^233,234^ The limited studies on nonhistone Klac have indicated its critical role in tumor, polymicrobial sepsis and retinopathy.

#### Protein lactylation in tumor

**Fig. 5:**
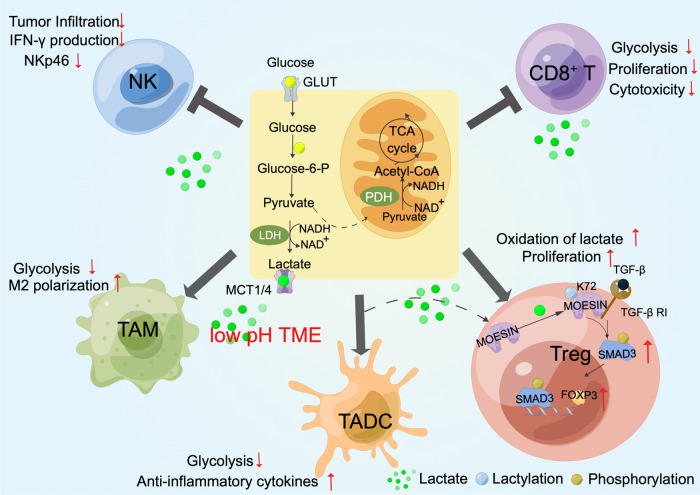
Lactate and protein lactylation contribute to the suppressive tumor immune microenvironment. Anaerobic glycolysis produces lactate in the cell cytoplasm, which is transported to the extracellular matrix (ECM) via MCT1/4, resulting in low pH tumor microenvironment (TME). The lactate in the ECM inhibits NK cells tumor infiltration and activity, reduces CD8^+^ T cells glycolysis, proliferation and cytotoxicity. Lactate also promotes the M2 polarization of macrophages and increases the release of anti-inflammatory cytokines from tumor-associated dendritic cells (TADCs). Besides, lactate enhances Treg cells function through promoting lactylation of MOESIN at the K72 residue in the cytoplasm, which upregulates TGF-β signaling via enhancing the interaction between MOESIN and TGF-βRI and increases expression of FOXP3

Based on the Warburg effect, the lactate concentration in tumor tissue is 5–20 times higher than in normal tissue (1.8–2.0 mM), which forms the microenvironment with low pH to promote tumor progression. In the previous study, histone Klac has been suggested to promote tumor development through promoting the immunosuppressive role of tumor-infiltrating myeloid cells or driving TAMs polarization to an M2-like phenotype. Recently, Gu et al., discovered that lactate enhances Treg cells through lactylation of MOESIN at the K72 residue in the cytoplasm, which upregulates TGF-β signaling via TGF-βRI and increases expression of FOXP3. MOESIN is primarily expressed in the cytoplasm, while TGF-βRI is mainly distributed on the cell membrane. The lactylation of MOESIN enhanced the interaction between MOESIN and TGF-βRI via providing an additional hydrogen bond. This study firstly indicated that Klac in nonhistone proteins plays a critical role in PPI and signal transduction. Besides, they found that patients responding to PD-1 treatment have lower levels of MOESIN lactylation than nonresponders, providing a novel idea of targeting Treg cells via anti-lactate approaches in enhancing tumor immunotherapy (Fig. 5).^235^

#### Protein lactylation in polymicrobial sepsis

As described above, lactate in the blood has been suggested as a biomarker of sepsis, which is a life-threatening disease characterized by organ dysfunction and dysregulation of inflammatory response.^236^ High mobility group box-1 (HMGB1) is associated with the severity and mortality of this disease.^237^ During polymicrobial sepsis, macrophages uptake extracellular lactate through monocarboxylic acid transporter (MCT), promoting HMGB1 Klac in the nucleus through a p300/CBP-dependent manner.^237^ The lactated HMGB1 will then enhance its acetylation by inhibiting the deacetylase SIRT1 and recruiting the HAT p300/CBP to the nucleus. The lactylated/acetylated HMGB1 in macrophages is then diffused to and accumulated in the cytoplasm and released through the exosome secretion pathway. This will in turn destroy endothelial integrity and increase vascular permeability, leading to endothelial cell barrier dysfunction and promoting sepsis development. Reducing lactate production in vivo can reduce HMGB1 levels and improve survival outcomes in patients with polymicrobial sepsis. In this study, the working mechanisms of Klac and the relationship between Klac and Kace are still indistinct. The molecular weight of HMGB1 is about 25 KD, which shuttles between cytoplasm and nucleus through passive transport. We can hazard guess that the protein polarity change caused by lactyl group might prevent HMGB1 from entering the nucleus.

### Protein myristoylation and its role in human diseases

Four kinds of protein lipidation, myristoylation (C14), palmitoylation (C16), prenylation (C15 or C20), and the glycosylphosphatidylinositol anchor, have been identified to be essential in regulating the structure and function of numerous proteins. Among them, myristoylation and palmitoylation are very important in the progress of many diseases.

Since the discovery of 3’,5’-cyclic adenosine monophosphate (cAMP) myristoylation in 1982, approximately 100 kinds of myristoylated proteins have been identified, including serine/threonine kinases, tyrosine kinases, substrates of kinases, phosphatases, regulators of vesicle transport, and signal transduction proteins.^238^ Although the ratio of myristic acid to total fatty acids is very small, proteins still utilize this kind of rare fatty acid as an N-terminal glycine or lysine modifier of new peptides due to its moderate hydrophobicity. The hydrophobic myristoyl group ensures the proper functioning and intracellular trafficking of proteins. For a long time, the myristol moiety is considered insufficient for protein-membrane associations unless additional membrane-affinity motifs, such as a stretch of basic residues and a second lipid modification. Until recently, Xiong et al. demonstrated that the electrically neutral N-terminal fragment of the protein kinase A catalytic subunit (PKA-c), in which myristoylation is the only functional motif sufficient for membrane association, providing a revised concept for the myristoylation working mode.^214^ Protein myristoylation has been reported in tumor, immune response, viral infection, and neurological disorders.^239^

#### Protein myristoylation in tumor

Protein N-myristoylation has been reported to promote tumor progression through regulating autophagic flux, protein affinity with cell membrane and protein degradation.

##### Myristoylation regulates autophagy by affecting protein affinity with lysosomes or mitochondrial membranes

AMP-activated catalytic subunit alpha-1 (AMPK) plays critical roles in the surveillance of mitochondrial damage and mitophagy induction.^240–242^ NMT1 mediates AMPKβ myristoylation to enhance its recruitment to damaged mitochondria. This in turn promotes the interaction of AMPK and the ATG16 complex and mitochondrial removal through autophagy, which helps to maintain cancer cell viability in ccRCC, lung cancer, and ovarian serous cystadenocarcinoma (OSC).^243^ In another two studies, researchers found that myristoylation of the lysosomal adaptor mTOR activator 1 (LAMTOR1) by NMT1 at G2 promotes its palmitoylation and anchoring to the lysosome, which increases mTOR activity to inhibit autophagy and promote the progression of lung cancer, bladder cancer, colorectal cancer, and cervical carcinoma.^244,245^

##### Myristoylation affects oncoprotein affinity with cell membranes and their signal transduction capacity

Src is a nonreceptor tyrosine kinase that acts as key mediators of cellular signal transduction and membrane binding. NMT1 regulates Src kinase myristoylation and phosphorylation, which allows the attachment of Src to the cytoplasmic membrane and mediates its kinase activity and cellular functions. Activation of SRC increases many downstream signaling pathways to facilitate tumor growth, angiogenesis, and metastasis in CRC and PCa.^246^ FGF/fibroblast growth factor receptor (FGFR) signaling is also regulated by myristoylation. For the activation of FGF/FGFR signaling, a scaffold protein called fibroblast growth factor receptor substrate 2α (FRS2α) must be recruited to initiate downstream signaling.^247^ Myristoylation of FRS2α is essential for the anchoring of FRS2α to the plasmatic membrane, which facilitates its binding with FGFR and helps to activate downstream phosphatidylinositol 3-kinase/AKT signaling and RAS/ERK signaling.^248^ Activated FGF/FGFR signaling ultimately promotes cell proliferation and migration in several cancer cell types.^249,250^ For AKT, myristoylation not only enhances its anchoring to the plasma membrane but also confers it to higher basal kinase activity, which upregulates aerobic glycolysis and fatty acid metabolism to promote tumor progression.^251,252^ Highly metastatic ovarian cancer cells have higher expression of the genes involved in fatty acid metabolism, especially acyl-CoA synthetase long-chain family member 1 (ACSL1), which contributes to an increase in phospholipids with relatively short FA chains, including myristic acid. Abundant myristic acid induces the myristoylation of protein kinase AMPK and Src, which in turn promotes ovarian cancer metastasis.^253^ These studies indicate that there may exist a positive feedback loop between lipid metabolism and myristoylation to accelerate tumor progression.

##### N-myristoylation affects oncoprotein degradation

N-myristoylation plays critical roles in boosting liver tumorigenesis, possibly by interfering with the balance between two categories of proteins, N-myristoylation downregulated proteins (NDP, including LXN, RPL29, and FAU) and N-myristoylation upregulated proteins (NUP, including AHSG, ALB, and TF), which oppositely control transformative phenotypes. The two types of proteins were revealed to be negatively and positively regulated by NMT1, respectively. Mechanistically, N-myristoylation influences the ubiquitination of NUP and NDP by affecting their binding with the E3 ubiquitin ligase HIST1H4H.^254^ Breaking N-myristoylation might be helpful to treat liver cancer.

#### Protein myristoylation in infectious disease

Recent studies have revealed that protein N-myristoylation is involved in host defense against viral and microbial infections.^255^ Here, we mainly discussed the former one. Protein N-myristoylation is necessary for viral invasion, assembly, intracellular host interactions and budding apart from a few exceptions.^256^ For example, the myristoylation of virus-encoded protein (VP0) in rhinoviruses and the gag or nef protein in HIV-1 is vital for viral replication, assembly, and infection^257–259^ The high cell membrane affinity or changed protein activity caused by the myristoyl moiety contributes a lot to these biological processes. As virus is lack of NMT1/2 to catalyze this modification, their proteins are myristoylated by host NMTs. NMT inhibitors such as IMP-1088 have shown pretty good effects on inhibiting virus assembly and replication.^258^ It is interesting and reasonable to study whether there is myristoylation in proteins of SARS-CoV-2, which may constitute a target in the development of COVID-19 therapeutic drugs.

#### Protein myristoylation in neurological disease

Parkinson’s disease (PD) is a chronic, progressive neurodegenerative disorder with no satisfied treatment strategy. Virus-delivered neurotrophic protein molecules within brain extracellular space to directly activate the intracellular signaling pathways is a promising therapeutic direction. Akt signaling maintains cell viability through antiapoptotic effects and mediates effects on axonal caliber, branching, and regeneration in neuron.^260,261^ As described above, myristoylated AKT could be recognized as its constitutive active form. In an in vivo study, Ries et al. demonstrated that transduction of myristoylated AKT via adeno-associated virus confers almost complete protection against apoptotic cell death in a highly destructive neurotoxin model.^262^

### Protein palmitoylation and its role in human diseases

Protein palmitoylation is a reversible dynamic process that was initially discovered in the 1980s. Similar to myristoylation, palmitoylation mainly acts as a lipid anchor that links proteins with specific membrane domains or lipid rafts, which helps signal transduction. In addition, S-palmitoylation is also reported to affect PPIs, protein stability, and protein aggregation. Over 300 proteins have been biochemically confirmed to be S-palmitoylated, and many of them function in cancer, COVID-19, neurological disease, immune disease, and cardiovascular disease.^263–270^

### Protein palmitoylation in tumor

#### Palmitoylation regulates tumor glycolysis

Glucose transporter (GLUT1) is a transmembrane protein that is responsible for the uptake of glucose into cells through facilitative diffusion and has been demonstrated to promote tumor progression in many cancer types.^271–274^ Plasma membrane (PM) localization is essential for glucose uptake by GLUT1. Researchers found that ZDHHC9 mediates the palmitoylation of GLUT1 at C207 and promotes its PM localization process, leading to a high level of glycolysis and glioblastoma (GBM) tumorigenesis.^275^

#### Palmitoylation regulates ER stress, and ER-controlled calcium (Ca^2+^) signal homeostasis

Disturbances in endoplasmic reticulum (ER) homeostasis activate the ER stress response, which promotes tumor cell apoptosis. Protein kinase R-like ER kinase (PERK), inositol-requiring enzyme 1a (IRE1a), and activating transcription factor-6 (ATF6) are three proteins that mediate the ER stress response.^276–278^ X-box binding protein 1 (XBP1) is a key transcription factor in the IRE1a signaling branch and is spliced by endoribonuclease and IRE1a to regulate protein folding, trafficking, and secretion, thus enhancing cell survival and ER homeostasis under ER stress.^279^ Researchers found that most PATs are upregulated in GBM. Inhibition of palmitoylation using 2-bromopalmitate (2-BP), cerulenin, and tunicamycin induces ER stress and cell death by inhibiting XBP1 palmitoylation. Ca^2+^ is a secondary messenger in cells and is mainly stored intracellularly in the ER to sustain a nanomolar cytosolic level. Malignant tumor cells usually exhibit a strong dependence on cytosolic Ca^2+^ for disease progression.^280–282^ Inositol 1,4,5-trisphosphate (IP3) interacts with IP3R in the ER membrane to release Ca^2+^ from the ER lumen.^283,284^ Fredericks et al. found that selenoprotein K (SELENOK) forms a complex with ZDHHC6 on the ER membrane and promotes IP3R palmitoylation at C56 and C849, leading to IP3R protein stabilization and Ca^2+^ release. By using spontaneous metastatic melanoma model with conditional SELENOK knockout mice, Michale et al. demonstrated that deleting SELENOK reduced tumor stemness, tumor growth and metastasis. This may be due to the inhibition of IP3R palmitoylation and reduced calcium flux necessary for tumor growth and metastasis.^285^

#### Protein palmitoylation in the regulation of oncogenic signaling

The protein palmitoylation of key oncoproteins helps to promote or inhibit tumor progression. The membrane protein claudin-3 (CLDN3) is critical for the formation and maintenance of tight junctions and is highly expressed in numerous cancers.^286^ ZDHHC12 mediates CLDN3 S-palmitoylation on three juxtamembrane cysteine residues (C181, C182, and C184), which contribute to its accurate localization in plasma membranes and protein stabilization, promoting ovarian cancer tumorigenesis. Palmitoylation also regulates G-protein-coupled protein activity.^287^ RAS proteins transduce extracellular signals from activated receptors at the PM to the nucleus, promoting cell proliferation, metastasis, and survival in ~30% of all human cancers. RAS family members, including NRAS, HRAS, KRAS4A, and KRAS4B, are highly homologous but distinct in the -CAAX motif at the C-terminus, which is subjected to PTMs to deliver RAS from the Golgi to the PM.^288^ Farnesylated and palmitoylated RAS proteins have a 100-fold higher affinity with membrane than that of only farnesylated Ras proteins. Researchers have demonstrated that the palmitoylation of oncogenic NRAS mediated by ZDHHC9 is essential for its PM localization and downstream signaling activation, promoting leukemogenesis.^289,290^ Epidermal growth factor receptor (EGFR) is an essential driver of oncogenic signaling. ZDHHC20 palmitoylates EGFR on the C-terminal domain (C1025) to promote its ubiquitination-mediated lysosomal degradation, thus upregulating p85 binding and phosphatidylinositol 3-kinase (PI3K) signaling and inhibiting Grb2 binding and MAPK signaling. By using a Kras^G12D^-induced spontaneous lung cancer mouse model and *Zdhhc20* knockout mice, researchers demonstrated that the ablation of ZDHHC20 or induction of EGFR^C1025A^ mutant inhibited tumorigenesis, which seems contradictory to the increased EGFR activation. They explained that EGFR palmitoylation appears to be necessary for PI3K-AKT-Myc signaling in lung cancer, revealing that ZDHHC20 inhibition represents a therapeutic vulnerability in the PI3K-AKT pathway.^291^ Melanocortin-1 receptor (MC1R) is a key protein in determining hair and skin pigments. MC1R mutations often results in the occurrence of red hair color and promotes melanoma tumorigenesis under ultraviolet (UV) irradiation.^292,293^ Researchers have demonstrated that palmitoylation of MC1R mediated by ZDHHC13 under UV irradiation helps to enhance DNA damage repair and inhibit melanoma genesis. This is the only study in which the palmitoylation of proteins was demonstrated to inhibit tumor progression.^294^

#### Protein palmitoylation in shaping antitumor immune responses

Many palmitoylated proteins have a close relationship with the immune system. In a recent review,^295^ the authors summarized the important immune signaling pathways associated with palmitoylation, including STING, NOD1/2, and JAK-STAT in cytokine signaling, T-cell receptor signaling, chemotactic GPCR signaling, apoptosis, phagocytosis, and endothelial and epithelial integrity. Studies about protein palmitoylation in the regulation of tumor immune microenvironment is also emerging, mainly focusing on PD-1/PD-L1 and IFN-γ signaling. PD-L1 was found to be palmitoylated at C272 by ZDHHC9 in breast cancer to enhance its stability. Disrupting PD-L1 palmitoylation by site-specific point mutation or inhibiting the expression of its PAT ZDHHC9 sensitized breast cancer cells to T-cell killing and thus repressed tumor growth.^296^ Another group formulated the same conclusion with more details in CRC. They found that the palmitoylation of PD-L1 increases its binding with PM and inhibits its ubiquitination-mediated lysosomal degradation, while the PAT is ZDHHC3 but not ZDHHC9 in CRC.^297^ The same group further illuminated the regulatory role of palmitoylation on PD-1 stability in tumor cells. Mechanistically, ZDHHC9 mediates PD-1 palmitoylation at C192 to promote its binding with recycling endosomes, thus preventing its lysosome-dependent degradation. They demonstrated that palmitoylation of PD-1, but not PD-L1, promoted mTOR signaling and tumor cell proliferation.^298^ IFN-γ belongs to the type II interferon family and is secreted by activated immune cells. IFN-γ signaling plays a critical role in antitumor responses, as it activates the Janus kinase (JAK) signal transducer and activator of transcription 1 (STAT1) pathway to induce the expression of classical interferon-stimulated genes that have key immune effector functions.^299^ recent work found that C122 palmitoylation in IFN-γ receptor 1 (IFNGR1) acts as a sorting signal for IFNGR1 lysosomal degradation mediated by AP3D1 in CRC. OPTN, which is lost in early-stage human colorectal cancer, interacts with AP3D1 to hinder its recognition of IFNGR1, thereby maintaining IFNGR1 stability and the integrity of downstream MHC-I signaling, which promotes antigen presentation to T cells and inhibits CRC progression (Fig. 3c).^300^ This work suggests that targeting IFNGR1 palmitoylation might be a new strategy for promoting immune checkpoint therapy in CRC.

### Protein palmitoylation in COVID-19

During the SARS-CoV-2 infection, it’s a key event that the spike (S) protein in the virus directly interacts with the receptor angiotensin-converting enzyme 2 (ACE2) in the host cell surfaces. Recently, two studies have separately reported the palmitoylation of both S protein and ACE2 protein. Both the C15 and cytoplasmic tail of S protein were palmitoylated by ZDHHC2, 3, 4, 5, 8, 9, 11, 14, 16, 19, and 20, which is critical for S‐mediated syncytia formation and SARS‐CoV‐2 pseudovirus particle entry.^301^ In another study, the authors discovered the presence of ACE2 in extracellular vehicles (EVs) which are determined by protein palmitoylation. The C141 and C498 residues on ACE2 are palmitoylated by ZDHHC3, which is critical for the membrane-targeting of ACE2 and their EV secretion.^302^ Besides, they built the engineered PM-ACE2-EVs via fusing the S-palmitoylation-dependent PM targeting sequence with ACE2 to bind with SARS‐CoV‐2, which blocks S protein interaction with ACE2 and protect host against SARS-CoV-2-induced lung inflammation. It’s reasonable to speculate that ZDHHC inhibitors might also be a potential therapeutic strategy in treating COVID-19, which might kill two birds with one stone.

### Protein palmitoylation in neurological diseases

**Fig. 6:**
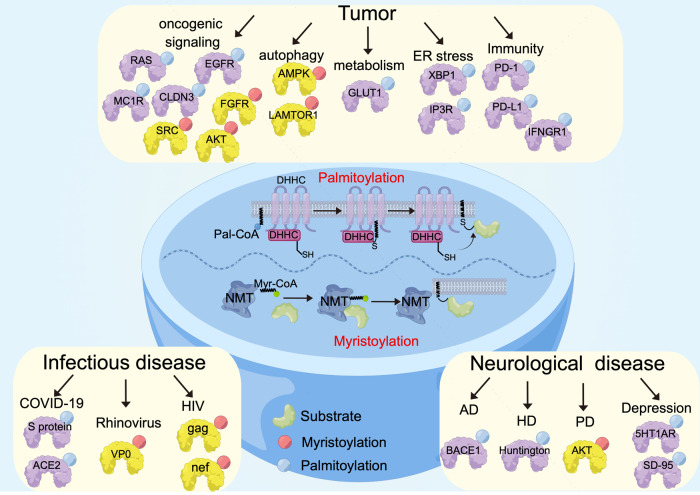
Protein palmitoylation and myristoylation within tumor, infectious diseases and neurological diseases in affecting protein-membrane binding. In the protein palmitoylation process, DHHC domain of ZDHHCs binds to the palmitoyl-CoA located in the membrane and undergoes autopalmitoylation, which is followed by a transfer of the palmitate group to the cysteine residue of the substrate protein, promoting the membrane localization of the substrates. The myristoylation process is similar with palmitoylation. First, NMT binds the fatty acid chain of myristoyl-CoA to form the myristoyl-CoA-NMT complex accompanied by substrate-binding pocket exposure, allowing the substrate protein to bind. Second, the NMT catalyzes N-myristoylpeptide formation through chemical transformation and releases the myristoylpeptide and CoA. These two kinds of PTMs promote tumor progression through regulating oncogenic signaling pathways, autophagy, tumor metabolism, ER stress, and tumor immune microenvironment. Besides, they are also critical to the infection of COVID-19, Rhinovirus and HIV, and play important roles in neurological diseases, including Alzheimer’s disease (AD), Huntington’s disease (HD), Parkinson’s disease (PD), and depression

Protein palmitoylation is an important process to regulate the physiological function of the brain, which plays important roles in AD, Huntington disease (HD) and depression. We have mentioned above that protein Ksuc of APP is critical to Aβ production. Here, researchers found that the palmitoylation of APP cleaving enzyme (BACE1) also positively determines the formation of Aβ, resulting in cognitive decline. This modification mainly decides dendritic spine localization and axonal targeting of BACE1 but not its enzymatic activity or protein stability.^303^ Besides BACE1, palmitoylation of γ-secretase complex, Fyn and flotillins/reggies have also been linked with AD progression.^303^ HD damages the corticostriatal circuitry in large part by impairing transport of brain-derived neurotrophic factor (BDNF), which is dependent on the protein of huntingtin.^304^ Pamitoylation of huntingtin by HIP14 (ZDHHC17) is essential for its trafficking and function.^305,306^ Restoring brain palmitoylation by inhibiting APT1 with the brain-permeable and selective molecule ML348 rescues neuron trafficking, which alleviate behavior and neuropathology of HD mice.^307^ Besides neurodegenerative disease, key proteins in depressive disorder is also found to be regulated by palmitoylation, including serotonin 1A receptor (5-HT1AR) and postsynaptic density protein 95 (SD-95).^308,309^ These studies provide promising clinical strategies for the treatment of neurodegenerative diseases and depression via manipulating palmitoylation of specific proteins. Protein palmitoylation and myristoylation both regulate tumor, infectious diseases and neurological diseases through affecting protein-membrane binding, therefore we summarized them in Fig. 6.

## Crosstalk between metabolism and acylation in human diseases

**Fig. 7:**
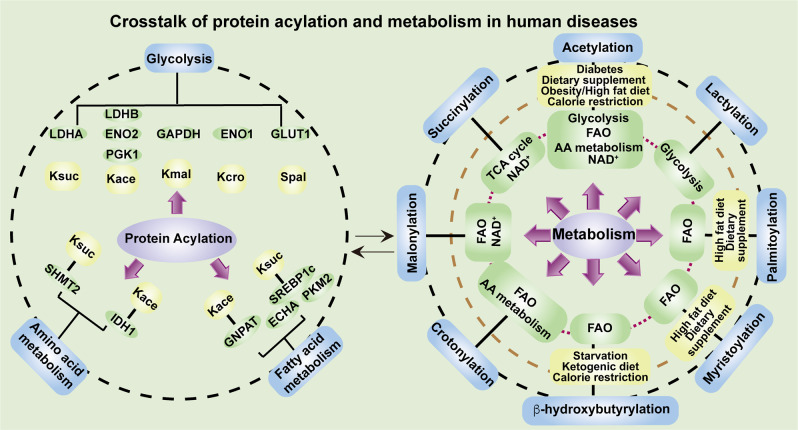
Crosstalk between protein acylation and metabolism in human diseases. Protein acylation on the metabolic enzymes or transporters always modulate human diseases through affecting glycolysis, fatty acid, and amino acid metabolism. As described in the left, LDHA succinylation, LDHB, ENO2, and PGK1 acetylation, GAPDH malonylation, ENO1 crotonylation and GLUT1 palmitoylation (Spal) can regulate glycolysis. SHMT2 succinylation and IDH1 acetylation regulate amino acid (AA) metabolism. Besides, GNPAT acetylation, SREBP1c, ECHA, and PKM2 succinylation influence fatty acid metabolism. From the view of disease, succinylation of LDHA, SHMT2, acetylation of ENO2, PGK1, IDH1, and GNPAT, crotonylation of ENO1 and palmitoylation of GLUT1 are critical in tumor; acetylation of LDHB and succinylation of SREBP1c is critical in hepatic steatosis; malonylation of GAPDH and succinylation of PKM2 is critical in inflammation; succinylation of ECHA is critical in cardiovascular disease. As described in the right, cell intrinsic and extrinsic metabolism conditions will in turn affect the eight kinds of protein acylations

Generally speaking, nearly all kinds of acylation respond to changes in metabolism, which suggests that acylation may help to integrate the responses to metabolic challenges. Both cell-intrinsic tumor metabolic reprogramming and cell-extrinsic factors, including dietary structure, dietary supplement and metabolic microenvironmental factors, such as obesity, diabetes and low pH, can regulate the acylation of proteins. Conversely, protein acylation reactivates and introduces changes to metabolism. Here, we summarized the topic from the following two aspects: how intrinsic and extrinsic metabolic state regulates protein acylation, and how protein acylation reciprocally regulates metabolism (Fig. 7). We mainly focus on tumor as examples.

### The intrinsic and extrinsic metabolic state on protein acylation

From the view of cell-intrinsic metabolic state, glycolysis, lipid β-oxidation, amino acid catabolism, and metabolism in TCA cycle all contribute to protein acylation. High cellular glucose uptake produces plenty of acetyl-CoA molecules and usually promotes protein acetylation especially in tumor.^101,310,311^ Lactate, a glycolytic product and a significant energy source also contributes a lot to protein acylation. Lactate could shuttle both intracellularly and intercellularly, establishing metabolic communication between different cell types in the microenvironment. For example, excessive lactate produced by both tumor and stromal cells can shuttle to Treg cells to induce lactylation of MOESIN at K72, which enhances tumor progression via sustaining Treg function.^235^ Lipid β-oxidation in mitochondria or the cytoplasm can enhance the abundance of acetyl-CoA, crotonyl-CoA, β-hydroxybutyryl-CoA, myristoyl-CoA and palmitoyl-CoA to promote the corresponding modifications. In addition, amino acid catabolism contributes greatly to acetyl-CoA and crotonyl-CoA for acetylation and crotonylation. High levels of α-KGDHC in the TCA cycle provide ample succinyl-CoA molecules for succinylation. Besides the supply of acyl groups, non-acyl metabolites have emerged as important regulators of acylation. The dinucleotide NAD^+^, a key metabolite that is involved in cellular electron transfer reactions, is a required cofactor for sirtuin. NAD^+^ mediates deacetylation, demalonylation and desuccinylation of target proteins.^312,313^ The NAD^+^/NADH ratio reflects the cellular redox homeostasis, suggesting that protein acylation could also act as a sensor to convert redox state to different cellular signaling. It is predictable that more details of metabolites in the regulation of nonhistone protein function will be elucidated to describe the complex interaction between acylation and metabolism.

Regarding extrinsic cell metabolism, diabetes and obesity or high-glucose/high-fat diet, ketogenic diet, starvation, and dietary supplement have been demonstrated to aggravate tumor progression by regulating protein acylation. Glycolysis produces acetyl-CoA, which is the donor of protein acetylation. Our recently published study has demonstrated that CRC patients with diabetes exhibit higher expression of p300 and TRIB3 than that of nondiabetic patients. Mechanistically, the high-glucose tumor microenvironment caused by diabetes mellitus could provide more acetyl-CoA and upregulate TRIB3 acetylation and protein stability, transforming metabolic signals to oncogenic signals.^101^ It seems reasonable to speculate that diabetes-associated hyperglycemia and high-glucose diet might promote tumor development by upregulating the protein acetylation described above. Lipid metabolism contributes greatly to protein acetylation, crotonylation, myristoylation, palmitoylation and β-hydroxybutyrylation. Obesity-associated high protein expression of leptin and TGF-β has been demonstrated to upregulate SMAD2 acetylation by inactivating ACC1 and promoting breast cancer metastasis.^314^ The donors of myristoylation and palmitoylation, myristic acid, and palmitic acid mainly exist in natural vegetable oil. Protein myristoylation and palmitoylation have been demonstrated to promote tumor progression by inhibiting autophagy and ER stress, activating numerous oncogenic signaling pathways and regulating immune response-related proteins. Ketogenic diet, fasting or calorie restriction could induce increased levels of β-OHB, which binds to HDAC1, HDAC2 and HDAC3 to inhibit their activities. Based on the HDACs inhibitory role, β-OHB can not only induce Kbhb modification, but also other types of acylation, such as acetylation.^315–317^ Therefore, limiting oil intake and preventing excessive hunger might help to prevent and even inhibit tumor growth. Mitochondrial adaptation is also crucial for the response to calorie restriction.^318^ In calorie-restricted mice, dramatic changes in acetylation were observed in liver mitochondria, with the majority changes of acetylation increasing. By contrast, in brown adipose tissue, calorie restriction led to markedly decreased mitochondrial acetylation. The strikingly different acetylation change between the two tissues may reflect the different physiological function of liver and brown adipose in response to calorie restriction. Dietary supplement also regulates protein acylation. L-carnitine, sphingosine-1-phosphateand inositol phosphates are also known to inhibit or activate the deacetylase activity of class I HDACs, resulting in histone acetylation and epigenetic regulation of gene expression.^319–321^

### Protein acylation in the regulation of metabolism

As described above, PGK1 is acetylated by PCAF at K323 to promote glucose uptake and liver tumorigenesis. ENO2 is deacetylated by HDAC3 at K394 to increase its activation and enhance glycolysis, resulting in PDAC metastasis. LDHA and ENO1, two critical enzymes of glucose anaerobic oxidation, are succinylated and crotonylated by CPT1A and CBP, respectively, to upregulate the production of lactate, aggravating tumor development. In addition, the glucose transporter GLUT1 is palmitoylated by ZDHHC9 to enhance glycolysis, providing energy for tumor progression. GNPAT is acetylated by ACAT1 at K128 to inhibit FASN degradation and enhance lipid synthesis. The IDH1 involved in glutamine metabolism is deacetylated at K224 to inhibit its enzymatic activity and the HIF1α-SRC transcription axis. SIRT5 catalyzes the desuccinylation of the serine metabolic enzyme SHMT2 to regulate one-carbon metabolism and promote tumor proliferation. c-Myc increases SDHA acetylation at K335 by promoting SKP2-mediated SIRT2 degradation to promote succinate production in the TCA cycle and H3K4me3 activation. In summary, protein acylation of metabolic enzymes or nutrient transporters always modulate the progression of different diseases by regulating glucose, fatty acid and amino acid metabolic reprogramming.

## Crosstalk among protein PTMs

### Crosstalk among protein acylations

Lysines can be posttranslationally modified by various types of acylations. A systematic study showed that a majority of succinylation sites in bacteria, yeast, and mouse liver were acetylated at the same position.^156^ It is predictable that one lysine site could be modified by several kinds of acylations. These modifications will form a competition with each other. Notably, except for lipidation, all the short-chain protein acylations share common regulatory enzymes, including writers, erasers and readers. For example, p300 almost mediates all the acylation of short-chain protein acylations. SIRT5 is simultaneously responsible for the deacylation of Kace, Ksuc, Kmal, and Kglu. However, the acylations of substrates are not often concurrently change with the regulatory enzymes. As described above, SIRT5 plays critical roles in cardiac function. Depletion of SIRT5 in transgenic mouse enhances protein succinylation significantly but has nearly no influence on protein acetylation.^40^ It is rational to guess that there are some preconditions for deciding enzymes’ selectivity in catalyzing their substrates, such as the different concentrations of acyl-CoA in various tissues, and the different binding affinity of enzymes with acyl-CoA.

### Crosstalk between acylation and other PTMs

Most mammalian proteins are modified by multiple PTMs, such as ubiquitination, phosphorylation, methylation and so on. These PTMs can reciprocally influence each other. Take acetylation as an example, Kace crosstalk with other PTMs can be principally classified into antagonistic or cooperative forms. For the antagonistic crosstalk, sometimes different modifications compete the same lysine sites with acetylation. The E3 ligase E4F1 and the acetyltransferase PCAF mediate mutually exclusive post-translational modifications of ubiquitination and acetylation of p53 at K320, respectively, in the determination of alternative cell fates: growth arrest or apoptosis.^322^ Also, the antagonistic crosstalk could happen at different amino acid sites. Activation by kinase-mediated phosphorylation and attenuation by phosphatase-mediated dephosphorylation are hallmarks of STAT1 signaling. Acetylation of STAT1 at K410 and K413 facilitate the association of p-STAT1 with phosphatases TCP45, allowing the dephosphorylation of STAT1 and adjust cytokine-induced gene expression rapidly and economically.^323^ It bears thinking about that under what situation the acetyl group could be added on p-STAT1 to induce it dephosphorylation and deactivation, but not maintaining an active state continuously.

Methylation-acetylation interplay in p53 is an example of cooperative crosstalk. In response to DNA damage, Set7/9 induces K372 methylation of p53, which subsequently enhances p53 acetylation and stabilization.^324^ Protein N-myristoylation of Src family kinases is critical to anchor the enzymes in the plasma membrane. In fact, it also acts as cooperative signal for Src family kinases phosphorylation. S13 of Lyn (Lyn-S13) could be phosphorylated by casein kinase 1γ (CK1γ) in a Lyn-G2 N-myristoylation-dependent manner at the Golgi during intracellular protein traffic.^325^ From these examples, we can get the impression that the assortment of different modifications on one protein is nonrandom and may occur in an ordered fashion, allowing precise control of protein stability or activity.

## Therapeutic opportunities for targeting protein acylation

Protein PTM has a strong relationship with many human diseases, including metabolic disease, cardiovascular disease, inflammatory and infectious disease, neurological disease, and tumor. Inhibitors or activators targeting protein PTMs have long been developed and showed great prospects in the treatment of those diseases, especially of cancer. For example, five inhibitors of HDACs have already been used to treat hematology in clinic including vorinostat (Merk, USA), romidepsin (Celgene, USA), panobinostat (Novartis, USA), chidamide (Chipscreen, China) and belinostat (Spectrum/Onxeo, USA).^326^ Many inhibitors of protein acylation against cancer have also been in clinical trials. Therefore, we mainly summarized the potential compounds targeting writers, erasers and readers of protein acylation in the treatment of cancer as follows. Chemicals targeting other diseases in the clinical trials are listed in Tables 2–4.

**Table 2:** Inhibitors of acylation writers entering the clinical trials

Inhibitor	Target	Mechanism	Clinical stage	Indications
CCS1477	p300/CBP	Binding to the bromodomain of p300/CBP.	Phase I, II	Advanced solid tumors
FT-7051	p300/CBP	Binding to p300/CBP bromodomain potently and selectively, which then blocks androgen binding and reduces AR activation.	Phase I	Metastatic castration-resistant prostate cancer
EP31670 (NEO2734)	p300/CBP BET	Targeting both BET and bromodomain of p300/CBP.	Phase I	Castrate-resistant prostate cancer, NUT carcinoma
PCLX-001	NMT1/2	Promoting the degradation of numerous myristoylated and nonmyristoylated BCR effectors, triggering apoptosis.	Phase I	B-cell non-Hodgkin lymphoma, advanced solid tumor

### Development of pharmacological agents for targeting protein acylation writers

Different subtypes of acyltransferases catalyze the acylation of histones and nonhistone proteins on specific amino acid residues. A potential role of the acylation modifications in the pathogenesis of cancer, cardiovascular disease, neurological disease, and immunological or infectious disease has been described. This indicates that specific inhibitors of acyltransferase are potential tools for pharmacological research and might find therapeutic applications.

#### Therapeutic opportunities for targeting writers of short-chain protein acylation

The acyltransferases of short-chain protein acylation are almost all overlapped and mainly belong to the KAT family except for the specific writers of Ksuc (CPT1A) and Kmal (PMAT1). CPT1A is not only a succinyltransferase but also a protein that catalyzes the rate-limiting step of fatty acid oxidation (FAO). ST1326, the only discovered inhibitor of CPT1A, has been demonstrated to inhibit the proliferation of acute myeloid leukemia. However, it achieves this effect mainly by regulating FAO but not protein succinylation.^162–164^ Besides, there is no compound targeting PMAT1. Therefore, we mainly summarized the inhibitors or activators targeting KAT families. The p300/CBP family is a main component of KATs, which play important roles in regulating the cell cycle, proliferation and differentiation and are highly expressed in colorectal cancer, lung cancer, breast cancer, liver cancer, prostate cancer, and different kinds of leukemia.^327–332^ Researchers have focused their efforts on targeting p300/CBP for a long time, and over 135 compounds have been discovered, which mainly target the HAT domain and BRD domain of these proteins.^333,334^ Here, we mainly review the compounds of high affinity and selectivity of p300/CBP with satisfactory anticancer effects.

Among the compounds that target the BRD domain of p300/CBP, an orally bioavailable, potent, and selective inhibitor of the p300/CBP bromodomain called CCS1477 was developed by the Cellcentric company. They demonstrated that CCS1477 inhibits cell proliferation in prostate cancer cell lines and decreases AR- and c-Myc–regulated gene expression.^331^ A phase I/II study to assess the safety, tolerability, pharmacokinetics and biological activity of CCS1477 in patients with metastatic castration-resistant prostate cancer (mCRPC) or advanced solid tumors was started in 2018. Another oral small molecule FT-7051 has been developed by the Forma Therapeutics Holdings, Inc. (FMTX), a clinical-stage biopharmaceutical company. It is designed to attach to the p300/CBP bromodomain potently and selectively, which then blocks androgen binding and reduces AR activation. This compound has shown an encouraging safety and effectiveness in the treatment of metastatic castration-resistant prostate cancer during the phase I clinical trial. EP31670 (NEO2734) is a novel potent oral dual BET and p300/CBP inhibitor, which shows more efficient tumor-killing ability than non-dual BET inhibitors in numerous solid tumors.^335^ This compound has also entered into the phase I clinical trial in the treatment of castrate-resistant prostate cancer and NUT carcinoma (Table 2).

In compounds that target the HAT domain, C646 and A485 both have high selectivity and antitumor effects. Bowers et al. and shrimp et al. obtained a pyrazolone small molecule compound (C646) through virtual screening, which has been demonstrated to inhibit tumor proliferation, promote tumor apoptosis, alter glucose metabolism, reduce tumor drug resistance and induce tumor immune response in different kinds of cancers.^336–343^ For example, in our study, C646 was confirmed to inhibit the K240 of TRIB3 and promote CD8^+^ T-cell recruitment in CRC tissues. The combination of C646 and PD-1/PD-L1 blockade therapy exhibited an increased antitumor effect compared to that of single immune checkpoint therapy.^101^ Researchers at the Abbvie company designed a class of compounds with the structure of spirindan hydantoin. After further optimization, they finally obtained compound A485 with nanomolar affinity, high selectivity, drug-like properties, and oral bioavailability.^344^ The compound has been demonstrated to have significant inhibitory activity in NSCLC, mantle cell lymphoma, multiple myeloma, non-Hodgkin’s lymphoma cells, and AR-positive prostate cancer cells.^345–347^ Similar with C646, A485 can enhance the tumor-killing effect of PD-L1 antibody.^348^ Mechanistically, it blocks the acetylation of histone H3 at the PD-L1 promoter and inhibits its transcription. In a recent study, researchers developed a proteolysis-targeting chimera (PROTAC) compound termed JQAD1 by linking A485 with the CRBN that selectively targets p300 for degradation. JQAD1 has been demonstrated to cause neuroblastoma cell apoptosis concurrent with MYCN downregulation.^349^ Compounds that target other KAT families are still in the early developmental stage and are not discussed here.

#### Therapeutic opportunities for targeting writers of protein lipidation

NMT and ZDHHC families are writers for protein myristoylation and palmitoylation. To date, NMT1, NMT2, ZDHHC2, ZDHHC3, ZDHHC6, ZDHHC8, ZDHHC9, ZDHHC12, ZDHHC13, and ZDHHC14 have been closely linked with most cancer types.^275,287,350–356^ The development of inhibitors targeting NMT and ZDHHC family to treat cancer is in progress.

The compound used as NMT inhibitors to combat tumors are still at the stage of laboratory research, including 2-hydroxymyristic acid 3, D-NMAPPD (also named B13), Tris-DBA palladium, IMP-366 (DDD85646), IMP-1088 and PCLX-001.^258,357–360^ Myristic acid analog 2-hydroxymyristic acid 3 was the first compound reported to inhibit NMT. B13 has been suggested to inhibit tumor progression by downregulating LAMTOR1, SRC and FRS2α myristoylation, as mentioned above.^244,246^ Tris-DBA palladium has been reported to have antitumor activity against melanoma. However, in a recent study, scientists found that N-myristoylation was unaffected by 2-hydroxymyristic acid 3, B13, and Tris-DBA palladium, and the tumor-killing ability involves some kind of off-target toxicity. IMP-1088, IMP-366, and PCLX-001 (which is orally bioavailable derivatives of IMP-366) have been identified as on-target and selective inhibitors of NMT.^359^ IMP-366 has been suggested to drive tumor apoptosis and reduce tumor growth by preventing mTORC1 activation and blocking lysosomal degradation. PCLX-001 has been demonstrated to inhibit the growth of hematological cancer cells in vitro and alleviate tumor progression in lymphoma murine xenograft models. Mechanistically, PCLX-001 promotes the degradation of numerous myristoylated and nonmyristoylated BCR effectors, triggering apoptosis. This potential compound has entered the phase I trial in treating B-cell non-Hodgkin lymphoma and advanced solid malignancies, and further tests will be performed^361,362^ (Table 2).

The development of anti-palmitoylation drug face huge hurdles. Presently, no potent and specific inhibitors against ZDHHC proteins have been discovered.^363,364^ 2-BP, cerulenin, and tunicamycin are PAT inhibitors that are extensively used in the literature, and 2-BP is the most common one. For example, these three compounds have been demonstrated to induce G2 cell cycle arrest and cell death in GBM cells through enhanced ER stress.^279^ In addition, tunicamycin (5, 10, and 25 μg/ml) and 2-BP (25, 50, and 150 μM) significantly decreased CSC sphere formation in the liver without affecting cell viability.^365^ 2-BP could also activate antitumor immunity in vitro and in mice bearing MC38 tumor cells. However, the nonspecificity of 2-BP limits its development into a drug. Efforts to screen for more selective inhibitors have been made, but this endeavor is difficult due to the extreme similarity of the PATs. Competitive inhibition is an effective approach for targeting specific enzymes. After identifying the palmitoylation motif of PD-L1 and PD-1, Yao et al. synthesized peptides that were derived from PD-L1 (MMDVKKCGIQDTNS-GFP) and PD-1 (Flag-AVICSRAARG-GFP), and these peptides exhibited a successful inhibitory effect that involved PD-1/PD-L1 palmitoylation and slowed tumor progression.^297,298^ Future studies are necessary to develop more selective PAT inhibitors.

### Development of pharmacological agents for targeting protein acylation erasers

Most of the protein acylations are dynamic, reversible, and highly regulated chemical reactions. HDAC family members regulate the homeostasis of protein acylation. Inhibitors of HDACs were used as anticancer drugs or even neuroprotectors by enhancing synaptic plasticity, learning and memory in a wide range of neurological disorders.

#### Therapeutic opportunities for targeting erasers of short-chain protein acylation

As described above, HDAC family are the common erasers for short-chain protein acylation, participating in tumor progression. Class I HDAC proteins are mainly distributed in the nucleus, among which HDAC1 and HDAC2 are highly expressed in lung cancer, breast cancer, gastric cancer, ovarian cancer, colorectal cancer, and pancreatic cancer.^366–370^ Class II HDAC proteins are mainly distributed in the cytoplasm and can shuttle from the cytoplasm to the nucleus, and HDAC4 and HDAC6 are highly expressed in liver cancer and oral squamous cell carcinoma, respectively.^371,372^ Class III contains members of the sirtuin family, which are usually responsible for the deacetylation of nonhistone proteins. SIRT5 and 7 have been demonstrated to promote tumor progression in lung and breast cancers. The only class IV HDAC member, HDAC11, has also been reported to promote lung cancer^373^ and hepatocellular carcinoma^374^ in recent years.

Targeting HDAC families is a novel therapeutic strategy against cancer. As described above, four products that target HDACs have been approved by the food and drug administration (FDA) of the USA, including vorinostat, romidepsin, belinostat, and panobinostat for the treatment of cutaneous T-cell lymphoma. Another product has been approved by China food and drug administration (CFDA) for the treatment of peripheral T-cell lymphoma—chidamide (tucidinostat), marking the first example of a “Made in China” drug. Except for chidamide, which is a selective inhibitor of class I HDACs and HDAC10, the other four compounds are all pan HDAC inhibitors. These compounds provide good antitumor effects on leukemia but unsatisfactory effects on solid tumors in the monotherapy. Therefore, clinical trials are currently being performed, in which the combination of HDAC inhibitors with chemotherapeutic drugs, kinase inhibitors or checkpoint inhibitors are being tested for treating solid tumors.^375,376^ Beyond the five compounds on the market, many novel HDAC inhibitors have entered clinical trials. For example, CRA-024781 (ABEXINOSTAT) with high selectivity of HDAC6 and 8, entinostat with high selectivity of HDAC1 and 3 and the pan HDAC inhibitor CI-994 have all entered the phase III clinical trial for the treatment of renal cell carcinoma, advanced breast cancer, and lung cancer.^377,378^ Many others are in the phase I–II clinical trials for the treatment of solid tumor or leukemia.^379–383^ Besides, some compounds showed potential effectiveness in treating neurological diseases, metabolic diseases or inflammatory and infectious disease and are tested in clinical trials (Table 3).

**Table 3:** Inhibitors of acylation erasers entering the clinical trials

Compound	Target	Mechanism	Clinical stage	Indications
Vorinostat	Pan HDACs	Inhibit the accumulation of acetylated histone and nonhistone proteins accumulation.	on the market	Cutaneous T-cell lymphoma
Romidepsin	Pan HDACs	Inhibit the activity of HDAC1 and 2, promoting cell apoptosis.	on the market	Cutaneous T-cell lymphoma
Belinostat	Pan HDACs	Inhibit the accumulation of acetylated histone and nonhistone proteins accumulation, inducing autophagy.	on the market	Cutaneous T-cell lymphoma
Panobinostat	Pan HDACs	Inhibit the accumulation of acetylated histone and nonhistone proteins accumulation, inducing autophagy and apoptosis.	on the market	Cutaneous T-cell lymphoma
Chidamide (tucidinostat)	HDAC class I, HDAC10	Induce the acetylation of H3 protein.	on the market	Peripheral T-cell lymphoma
Resminostat	Pan HDACs	Inhibit the activity of HDAC1, 3, and 6.	Phase IV	IgA nephropathy
Abexinostat	Pan HDACs	It has a higher selectivity of HDAC6 and 8.	Phase III	Renal cell carcinoma
Givinostat	Pan HDACs		Phase III	Duchenne muscular dystrophy (DMD)
Entinostat	HDAC1, 3	Inhibit the activity of HDAC1/3 and induce cell autophagy and apoptosis.	Phase III	Advanced breast cancer
CI-994	Pan HDACs	Inhibit the activity of HDAC1, 2, 3, and 8 and cell proliferation.	Phase II	Pancreatic cancer, multiple myeloma
			Phase III	Lung cancer
Domatinostat (4SC-202)	HDAC class I	Induce hyperacetylation of H3 histone proteins.	Phase II	Merkel cell carcinoma
Tinostamustine (EDO-S101)	HDAC class I, II	Induce acetylation of histone or nonhistone proteins.	Phase I, II	Numerous solid tumors
4-Phenylbutyric acid	Pan HDACs	Inhibit histone deacetylase and ER stress induced by SiNPs in RAW264.7 cells.	Phase I	Lymphoma, adult solid tumor
			Phase I	Neurodevelopmental disorder
KA2507	HDAC6	Inhibit HDAC6 activity and shows antitumor activity and immunomodulatory effects in preclinical models.	Phase I	Solid tumor
CXD101	HDAC class I	Inhibit the activity of HDAC1, 2, and 3.	Phase I	Advanced cancer
Tasquinimod	HDAC4	It works by allosteric inhibition of HDAC4 signaling	Phase I	Multiple myeloma
AR-42	Pan HDACs	Downregulate histone acetylation and Akt signaling.	Phase I	Recurrent plasma cell myeloma, adult acute myeloid leukemia
Citarinostat	HDAC6	Inhibit the activity of HDAC6 and cell proliferation.	Phase I	Smoldering multiple myeloma
SIRT2104	SIRT1	A selective SIRT1 activator involved in the regulation of energy balance.	Phase I	Colitis
			Phase II	Diabetes mellitus, type 2
			Phase II	Psoriasis

#### Therapeutic opportunities for targeting erasers of protein lipidation

Although in most cases protein palmitoylation and myristoylation contribute to tumor progression, they also exhibit antitumor activity in some cases. It might be more effective to prevent depalmitoylation or demyristoylation in such specific cases. SIRT6 and IpaJ are erasers of lysine and N-terminal glycine myristoylation, however, no specific inhibitors targeting these erasers to regulate myristoylation have been developed yet. Chemicals targeting APT, PPT, and ABHD families in depalmitoylation are still in their early stage. Palmostatin B (PB) and palmostatin M (PM) are APT inhibitors that covalently modify and inactivate the serine residue in the active site but lack specificity between APT1 and APT2.^384,385^ PB treatment was found to block the proliferation of AML blasts from a mouse model that harbored oncogenic N-Ras.^385^ Using fluopol-activity-based protein profiling (ABPP), researchers have identified another APT inhibitor chemotype with a piperazine amide motif and discovered two compounds named Inhibitor 21 and Inhibitor 1, which selectively target APT1 and APT2.^386^ Beyond inhibition of ATP1/2 to inhibit tumor development, inhibition of PPT1 mediated depalmitoylation may lead to more direct toxic effect in cancer cells. The natural product didemnin B and dimeric quinacrine compounds—DQ661 have been demonstrated to inhibit solid tumor growth via inhibiting PPT1 and disrupting lysosome function.^387,388^ Except for APT and PPT, inhibitors of the ABHD family are also being developed. A potent and selective covalent inhibitor of ABHD17–ABD957 has been demonstrated to inhibit the proliferation in N-RAS-mutant cancer.^389^

### Development of pharmacological agents for targeting protein acylation readers

BET proteins, especially BRD3 and BRD4, are the readers of nonhistone protein acetylation, which are critical in a wide spectrum of human diseases. More and more evidence supported the use of BETi in treating cancer, metabolic, inflammatory, neurologic, cardiovascular, and musculoskeletal diseases. Over 50 BRD3/BRD4 inhibitors have been developed after the discovery of a first series of compounds in the treatment of cancer, including JQ1, I-BET151, and I-BET762.^390–394^ These inhibitors can be categorized into two types: (1) inhibitors binding to BD1 or BD2 domains of BRD3/4 and interrupting the enzymatic activity, such as PLX51107 and apabetalone, which have entered the phase I or II clinical trials in the treatment of tumor, COVID-19 infection, cardiovascular disease or diabetes (Table 4).^395–397^ (2) the PROTAC proteins promoting the degradation of BRD proteins, such as BRD4 ligand-Linker Conjugate 1, PROTAC BRD4 degrader-5/15, AT6, OARV-771.^398,399^ BET inhibitors in monotherapy have shown active effects, while combination studies might have a big impact to improve BET inhibition activity and reduce toxicity.

**Table 4:** Inhibitors of acylation readers entering the clinical trials

Inhibitor	Target	Mechanism	Clinical stage	Indications
PLX51107	BRD3, BRD4	Blocking some of the enzymes needed for cell growth.	Phase I	Acute myeloid leukemia
			Phase I, II	Acute graft versus host disease, steroid-refractory graft versus host disease
OTX015/Birabresib	BRD3, BRD4	Inhibiting the binding of BRD3 and BRD4 to AcH4 and downregulate c-Myc expression.	Phase I	Acute myeloid leukemia
BI 894999	BRD4 (BD1), BRD3 (BD2)	Inhibiting the binding of BRD4 and BRD3 with acetylated histone proteins.	Phase I	Neoplasms, NUT carcinoma
Apabetalone	BRD4 (BD2)	Displacing BET proteins from chromatin	Phase I, II	Pulmonary arterial hypertension
			Phase II, III	COVID-19
			Phase I, II	Dyslipidemia, atherosclerosis
			Phase III	Diabetes mellitus, type 2

## Conclusions and future perspectives

With the discovery of PTMap, a sequence alignment software for unrestricted, accurate, and full-spectrum identification of PTM sites, research on protein acylation have undergone unprecedented development in the past 10 years. Many novel short-chain protein acylations were identified, which have been deeply studied in various diseases. As all the acylation donors come from metabolites, protein acylation is helping researchers to understand the regulation mechanism of metabolism in both physiological and pathological conditions, providing numerous drug targets in tumor, cardiovascular diseases, metabolic diseases, inflammatory and infectious diseases, etc. This exciting area has become a hot topic in basic research and will bring so many opportunities in translational medicine, however, there are still many challenges to be solved.

### Protein acylation in basic research

A lot of efforts have been made in basic research to search for new or specific protein acylations on critical proteins, identify animo acid sites of acylation and study their biological function in different diseases. Several questions or obstacles are existed focusing these work.

1. New approaches besides analytical chemistry of identifying protein acylation are still being needed. Although effective, inaccuracies in spectral matching often result in false-positive identifications in conventional PTM identification via MS, which is based on the fixed mass shift of the modified peptide. The development of complementary approaches is in urgent need. (A) Exploiting signature MS/MS ions to validate the presence of PTMs is invaluable, such as cyclic immonium ion of lactyllysine. (B) Although several pan acylation antibodies were developed and widely used to examine the acylation of proteins in specific cells or tissues, some antibodies targeting specific acylations are still in need, such as the detection antibody for protein palmitoylation. (C) Techniques for the enrichment of acylated peptides may also be useful. For example, scientists reported an enrichment approach based on a novel magnetic microsphere modified with 2,2′-dithiodipyridine (Fe_3_O_4_/SiO_2_-SSPy microsphere), which demonstrated remarkable enrichment selectivity and sensitivity for palmitoylated peptide, enabling a global annotation of protein palmitoylation for complex biological samples.^400^
2. The methods for verifying protein acylation modification sites need to be carefully considered. Before the discovery of several novel short-chain protein acylations, K to arginine (R) mutation in the substrate was thought to mimic the lysine deacetylation, besides, K to glutamate (E) mutation was thought to mimic the lysine acetylation. However, K-to-E mutation is also considered as a mimic of the succinylated state. Moreover, other PTMs, such as ubiquitination and sumoylation can also occur at lysine residues. That is to say, in some conditions researchers should reconsider whether such mutation really mimics the desired acylation or whether the mutation affects other PTMs of the interest protein, especially in functional study. Although more and more acylation sites were experimentally characterized, the regulatory enzymes for most of sites remain to be dissected. In contrast with labor-intensive and time-consuming experiments, computational prediction of acylation sites from protein sequences based on machine learning is much more helpful to generate highly useful information for further experimental consideration. Several online tools for protein acylation prediction are available now.^401–403^ We have reason to believe that more and more bioinformatic tools will constantly emerge, which will accelerate our comprehensive understanding of protein acylation. Combining of protein acylation prediction and specific PTMs antibody might be helpful for the determination.
3. The donors of several protein acylations have not been found in the human body yet, but from the microbiome in the gastrointestinal tract, including hydroxyisobutyrate, propionate, and butyrate. This might uncovered a new relationship between host and microbiome.

### Protein acylation in translational medicine

The discovery of molecular signatures that enables early diagnosis, accurate prognosis and personalized therapy is valuable in clinic, especially in the treatment of cancer. Protein acylation is a dynamic process and has close relationship with tumor, which sheds lights on this valuable requirement. For example, the propionylation level of histone H3K23 in U937 leukemia cells is at least six-fold higher than in non-leukemia cell lines. While, during monocytic differentiation, the propionylation level in U937 cells decreased remarkably, indicating that the initial hyperpropionylation in U937 cells might be a stage-specific marker in leukaemogenesis.^404^ In another study, authors found that higher levels of H2BK120, H3.3K18, and H4K77 acetylation in liver cancer tissues were significantly associated with worse prognosis, such as poorer survival and higher recurrence in an independent clinical cohort of HCC patients.^405^ Similarly, nonhistone acylations also demonstrate diagnostic or prognostic potential. High level of acetylated IDH1 at K224 are significantly correlated with advanced tumors, metastasis and reduced survival in CRC.^406^ Nonhistone acetylation also shows diagnostic potential for infectious disease. Acetylated K676 of transforming growth factor–beta-induced protein (TGFBIp) was consistently elevated in the blood of patients with SARS-CoV-2 pneumonia, especially in patients in the intensive care unit.^153^ In this study, treatment with TGFBIp neutralizing antibodies suppressed the cytokine storm, suggesting that K676 acetylation of TGFBIp can be used not only as a diagnostic marker, but also a indicator for TGFBIp neutralizing antibody therapy against SARS-CoV-2 pneumonia. For the neurodegenerative disease, succinylated Aβ and tau are closely associated with the disease state of AD,^173^ providing new molecular diagnostics and potential therapeutic targets. However, several issues are still waiting to be solved in the translational medicine of protein acylations.

1. Large-scale proteomic screening for the acylation of specific proteins in the peripheral blood, fecal or even saliva of patients is crucial to provide more early and convenient diagnostic markers or therapeutic targets for different types of diseases. For this purpose, developing technologies with high specificity and sensitivity to distinguish diverse acylations in a complex system is an urgent issue to be solved.
2. The close relationship of protein acylation and human diseases has rendered it as an attractive therapeutic target. However, there are two limitations in this field. First, the specificity of the inhibitors is generally low. For example, the HDAC inhibitors on the market are almost pan HDACi, producing lots of side effects. Second, the compounds mainly target acyltransferases and deacylases, which have broad influences on downstream substrates. As these enzymes usually play complex roles in regulating different substrates and may act as oncogenic proteins or tumor suppressors in a cancer-type-dependent manner, strategies that target specific protein PTMs are in need. The α-helical interfering peptides might be good candidates to interrupt the association of enzymes with their substrates, providing a more accurate regulation for protein acylation. In addition to the drug therapy, dietary patterns, such as ketogenic diet and restriction of glucose or long-chain fatty acids intake, are also important for the prevention and therapy of human diseases through regulating protein acylation. On this road, the great challenge remains of spatially and timely guiding such interventions to achieve disease-specific outcomes without compromising the responses of healthy cells.
